# Prevalence and risk factors of viral hepatitis and HIV among people experiencing homelessness in Germany based on a nationwide study

**DOI:** 10.1038/s41598-025-18552-3

**Published:** 2025-09-17

**Authors:** Fabian Heinrich, Tsz Lun Ernest Wong, Wiebke Graf, Katharina Dost, Anna Brennecke, Veronika Kowalski, Victoria van Rüth, Stefanie Iwersen-Bergmann, André Hajek, Hans-Helmut König, Thomas Renné, Thomas T. Brehm, Susanne Pfefferle, Julian Schulze zur Wiesch, Maura Dandri, Martin Aepfelbacher, Klaus Püschel, Benjamin Ondruschka, Marc Lütgehetmann, Franziska Stallbaum

**Affiliations:** 1 Institute of Legal Medicine, University Medical Center Hamburg-Eppendorf, Hamburg, Germany; 2 Department of Medical Statistics, London School of Hygiene and Tropical Medicine, London, UK; 3 Section of Ophthalmology, Twin Research and Genetic Epidemiology, King’s College London, London, UK; 4 Department of General, Visceral and Thoracic Surgery, University Medical Center Hamburg-Eppendorf, Hamburg, Germany; 5 Department of Health Economics and Health Services Research, University Medical Center Hamburg-Eppendorf, Hamburg, Germany; 6 Institute of Clinical Chemistry and Laboratory Medicine, University Medical Center, Hamburg-Eppendorf, Hamburg, Germany; 7 Center for Thrombosis and Haemostasis, Johannes Gutenberg University Medical Center, Mainz, Germany; 8 I. Department of Medicine, University Medical Center Hamburg-Eppendorf, Hamburg, Germany; 9 Department of Clinical Infectious Diseases, Research Center Borstel, Leibniz Lung Center, Borstel, Germany; 10 Institute of Medical Microbiology, Virology and Hygiene, University Medical Center, Hamburg-Eppendorf, Germany; 11 German Center for Infection Research (DZIF) Partner Site, Hamburg-Lübeck-Borstel-Riems, Heidelberg, Germany

**Keywords:** Infectious diseases, Viral hepatitis, Vaccination, Homeless, Incarceration, Infectious diseases, Viral hepatitis, Epidemiology, Risk factors, Epidemiology

## Abstract

People experiencing homelessness (PEH) are at risk of contracting and transmitting infectious diseases. Data on PEH blood-transmitted virus prevalence and vaccination coverage is needed to design targeted interventions. A nationwide multicentre cross-sectional study of PEH was conducted. Clinical data were collected through questionnaire-based interviews, and blood samples were tested by serology and qPCR. Latent class analysis (LCA) identified subgroups of PEH, while univariable regressions identified risk factors of viral hepatitis. 643 PEH from four metropolitan areas in Germany were included. LCA revealed national short-term, long-term and international short-term PEH subgroups. The prevalence of anti-HAV-IgG, anti-HBc, anti-HBsAg, anti-HCV, anti-HEV and anti-HIV was 44% (95%CI: 39–48%), 17% (95%CI: 14–21%), 25% (95%CI: 21–29%), 18% (95%CI: 15–22%), 29% (95%CI: 25–33%) and 0.7% (95%CI: 0.2–1.7%), respectively. Active HBV and HCV infection was detected in 1.4% (95%CI: 0.7–2.7%) and 12% (95%CI: 9.6–14%), respectively. Univariable logistic regression revealed PEH with former imprisonment had 13.24 times the odds of active or past HCV infection (95%CI: 6.28–27.90) that individuals without had. This study shows the high acquisition and transmission risks for HCV/HIV among PEH. Low vaccination coverage for HAV/HBV calls for revising vaccine recommendations. High HCV infection risk associated with imprisonment highlights the need to address health disparities faced by incarcerated individuals.

**Supplementary Information:**

The online version contains supplementary material available at 10.1038/s41598-025-18552-3.

## Introduction

According to the Global Hepatitis Report 2024, approximately 1.3 million peopledied worldwide from viral hepatitis in 2022. The World Health Organization (WHO) has defined the goal of eliminating, or at least substantially reducing, chronic viral hepatitis by 2030^1^. In 2016, Germany introduced the ‘BIS 2030’ strategy to achieve this objective at the national level^2^. Given the current trajectories, this aim will be missed globally^3^. From a public health perspective, people experiencing homelessness (PEH) may represent a reservoir for viral hepatitis, hindering its elimination^4^. PEH are an extremely heterogeneous and vulnerable population with increased somatic and psychiatric morbidity and mortality compared with housed individuals^5^. Living in crowded shelters and having limited access to hygienic or preventative measures poses PEH with a high risk of contracting and transmitting infectious diseases. The major routes of transmission differ among pathogens. Hepatitis A virus (HAV) and hepatitis E virus (HEV) are transmitted through the faecal-oral route, whereas the hepatitis B virus (HBV), hepatitis C virus (HCV), and hepatitis D virus (HDV) are transmitted via parenteral routes. Likewise, the human immunodeficiency virus (HIV) is transmitted parenterally. Accordingly, established risk factors for viral hepatitis include homelessness itself and related factors often observed among PEH, such as drug use, migration history, and lower socioeconomic status^3^.

In the United States of America (USA), homelessness has been defined as an indication for HAV vaccination, whereas in Germany, the clinician’s decision to screen, vaccinate, and treat viral hepatitis is based on individual risk assessment. For example, HAV and HBV vaccinations are recommended for men who have sex with men (MSM), people living in psychiatric facilities, and individuals using drugs intravenously^6^. However, identifying high-risk behaviours requires thorough anamnesis and trust between patients and physicians. People experiencing homelessness often lack access to the public healthcare system, are highly mobile, and face language barriers, along with mistrust and non-compliance. These factors make it difficult to establish a successful physician–patient relationship^7^. In Germany, communities offer specialised low-threshold medical care for PEH, theoretically allowing the implementation of targeted interventions to increase vaccination rates and treat individuals with viral hepatitis. It is estimated that by 2022, as many as 607,000 individuals will experience homelessness, with up to 50,000 people living without shelter in Germany^8^. However, data on the prevalence and risk factors of viral hepatitis in PEH in Germany and other European countries are scarce and are often only generated in single-centre studies^9,10^. Therefore, the relevance of action may be underestimated, and interventions may not reach the most in need.

The National Survey on Psychiatric and Somatic Health of Homeless Individuals (NAPSHI) is a nationwide cross-sectional study conducted in Germany in 2021. The NAPSHI acquired virological data on the infection status of PEH with HAV, HBV, HCV, HDV, HEV, and HIV. Additionally, detailed information on sociodemographics, risk behaviours, and health status were collected through structured interviews and laboratory tests. The primary aim of the current study was to determine the prevalence of viral hepatitis and HIV and to identify subgroups with similar risk factor profiles. These findings can help inform policymakers and public health experts about the issue, as well as identify PEH groups that would benefit from targeted interventions.

## Methods

### Study design

The National Survey on Psychiatric and Somatic Health of Homeless Individuals (NAPSHI) was conducted between July and September 2021 in four German metropolitan areas: Hamburg, Frankfurt am Main (Frankfurt am Main, Mainz, and Wiesbaden), Leipzig (Leipzig and Halle), and Munich (Munich and Augsburg). Municipal authorities were contacted to obtain information from institutional representatives of public spaces, shelters, lodging houses, drug aid facilities, women’s shelters, and medical practices that offer specialised care forpeople experiencing homelessness. The authorities shared the contact details of 40 support facilities for PEH that had agreed to participate (n = 15 for the Hamburg metropolitan area, n = 9 for Frankfurt am Main metropolitan area, n = 6 for Leipzig metropolitan area, and n = 10 for Munich metropolitan area). Two weeks before enrolment, these sites received study information and materials for advertising purposes. A study team, comprising medical students and doctors, visited each study site. Both staff members and members of the study team approached visitors to the care facility and invited them to take part in the study.

The inclusion criteria were a lack of permanent residence (> 7 days), age > 18 years, and capacity to provide informed consent. Pregnant individuals were excluded from this study to avoid additional blood draws. Questionnaire-based interviews were offered to collect demographic, somatic, and psychiatric health data. Members of the study team conducted interviews. The questionnaires were available in German, English, Russian, Polish, and Bulgarian, with additional translation services and assistance from professionals and peers provided to facilitate understanding of the questions. Additionally, a short clinical exam was conducted, including weight and height measurements. Further details can be found in the study by Bertram et al. . Venous blood samples were collected by trained medical professionals. Serum blood samples were centrifuged, stored, and transported to the Institute of Medical Microbiology, Virology, and Hygiene at the University Medical Center Hamburg-Eppendorf, Hamburg, Germany, at 4 °C.

The study protocol conformed to the ethical guidelines of the 1975 Declaration of Helsinki, as reflected in the a priori approval by the institution’s human ethical committee (Hamburg Chamber of Physicians, application number PV7333). The study was conducted in accordance with local legislation and institutional requirements. Written informed consent was obtained from all patients included in the study. The patients had to provide separate consent for examining notifiable diseases. For this purpose, names and contact details were collected. If a participant was diagnosed with HIV, HBV, or HCV, both the participant and their trusted medical professional were contacted immediately. They were provided with original lab results and written information about local treatment facilities in the metropolitan area. An allowance of 5€/30 min was offered as compensation. The STROBE cross-sectional checklist was used to write the reports.

### Data processing

Each questionnaire was transcribed into an Excel spreadsheet by two research assistants. Subsequently, Excel spreadsheets were imported into a statistical programme for further data processing. All variables were inspected using descriptive statistics. Continuous variables were examined for implausible values by examining summary statistics and histograms. Categorical variables were checked for implausible values using tables. When implausible values were present, the first step was to verify if they resulted from transcription errors. If implausible values persisted, values were recoded as missing.

### Virological analysis

#### Serology and molecular diagnostics

All samples were analysed using commercial CE IVD-compliant assays in an accredited diagnostic laboratory at the Institute of Medical Microbiology, Virology, and Hygiene at the University Medical Centre Hamburg-Eppendorf. For anti-HAV IgG, anti-HCV, HBsAg, and anti-HIV antibodies, the Alinity I system (Abbott, Wiesbaden, Germany), and for anti-HEV IgG and anti-HDV, the LIAISON XL system (Diasorin, Saluggia, Italy) were used. Anti-HCV-positive samples were confirmed using HCV IgG line blot assay (CarL; Mikrogen, Neuried, Germany). HBsAg-positive samples were confirmed using an HBsAg confirmation assay (Alinity I System, Abbott). Furthermore, HBsAg-positive samples were tested for anti-HDV (LIAISON XL, Diasorin). Samples with reactive HIV screening tests were confirmed using HIV 1/2 IgG line blotting (CarL; Mikrogen). To detect and quantify HBV, HCV, and HIV viral loads, quantitative (RT-) qPCRs were performed using a fully automated system (Cobas 6800; Roche Diagnostics, Mannheim, Germany). For HDV, HEV, and rat HEV (Rocahepevirus ratti), laboratory-developed quantitative assays were performed on the utility channel of the Cobas 6800 system, as described previously ^11^.

#### Disease definition

Active disease was defined according to the current guidelines. Briefly, active HBV infection was defined as a positive HBsAg screening assay confirmed by an independent HBsAg neutralisation assay. Active disease for HCV, HEV and ratHEV was defined as the presence of the respective viral RNA. For the diagnosis of HIV, a positive HIV screening assay and a reactive HIV IgG blot result, and/or HIV 1 RNA > 1000 copies/ml were needed.

### Statistical analysis

Categorical variables are presented as numbers and proportions. Categorical variables were examined, and categories with less than 10% of observations were merged with other categories where clinically appropriate. Continuous variables were summarised as mean (SD) or median (IQR), as appropriate. Continuous variables were inspected for approximate normality using Q-Q plots. All variables were examined to determine the number and proportion with missing data. Exact 95% confidence intervals were calculated using the binomial distribution.

A generalised linear model with a binomial outcome distribution and its canonical link function was employed to examine risk factors for hepatitis and HIV infection. Acute or past viral hepatitis and HIV infection status served as dependent variables. The independent variables were included on a clinical basis. Continuous variables were included in a linear functional form. Firth’s bias correction addressed substantial data separation due to the small number of events in the HIV infection model.

Latent class analysis (LCA), an unsupervised machine-learning technique, was used to identify subgroups of individuals with similar demographic and risk behaviour profiles within the PEH cohort in Germany. The LCA was performed as described by Sinha and colleagues ^12^. First, variables were selected for LCA based on their plausible epidemiological or biological associations with the risk of infection by each hepatitis virus. See Tables 1 and 2 for variables used in LCA modelling. The most appropriate number of LCA classes was then selected based on model fit statistics, i.e., Bayesian Information Criteria (BIC), Akaike Information Criteria (AIC), and class separation using entropy. Besides considering model fit criteria, the number of LCA classes was evaluated for its epidemiological and clinical relevance before selecting the final model. The distribution of variables between the classes was examined to identify subgroup characteristics. More details on the steps involved in LCA are provided in Supplementary Material. Next, logistic regression was used to compare the odds of active and past hepatitis or HIV infection between classes, to establish whether the classes obtained from LCA had different risks of hepatitis.

**Table 1:** Sociodemographic characteristics of people experiencing homelessness by their most probable class assignment from latent class analysis.

	Total (N = 643) Median (IQR) and number (%)	Class 1 (N = 275) Median (IQR) and number (%)	Class 2 (N = 166) Median (IQR) and number (%)	Class 3 (N = 202) Median (IQR) and number (%)
Age, years	43·0 (35.0 to 53.0)	42.0 (35.0 to 52.0)	50.0 (40.0 to 55.0)	38.0 (32.0 to 46.0)
Sex				
Female	105 (17.0%)	52 (19.4%)	30 (18.8%)	23 (12.0%)
Male	514 (83.0%)	216 (80.6%)	130 (81.2%)	168 (88.0%)
Educational attainment				
No degree	107 (18.0%)	25 (9.5%)	36 (23.1%)	46 (26.3%)
School education	276 (46.5%)	144 (54.8%)	61 (39.1%)	71 (40.6%)
Higher education	211 (35.5%)	94 (35.7%)	59 (37.8%)	58 (33.1%)
Marital status				
Single	401 (67.2%)	192 (74.7%)	98 (61.6%)	111 (61.3%)
Married	69 (11.6%)	18 (7.0%)	15 (9.4%)	36 (19.9%)
Divorced/widowed	127 (21.3%)	47 (18.3%)	46 (28.9%)	34 (18.8%)
Characteristics of homelessness				
City of enrolment				
Frankfurt	151 (23.5%)	61 (22.2%)	36 (21.7%)	54 (26.7%)
Hamburg	200 (31.1%)	78 (28.4%)	68 (41.0%)	54 (26.7%)
Leipzig	101 (15.7%)	75 (27.3%)	15 (9.0%)	11 (5.4%)
Munich	191 (29.7%)	61 (22.2%)	47 (28.3%)	83 (41.1%)
ETHOS classification				
Roofless	337 (57.5%)	133 (52.2%)	94 (60.3%)	110 (62.9%)
Homeless	249 (42.5%)	122 (47.8%)	62 (39.7%)	65 (37.1%)
Time of homelessness, months	18.0 (5.0 to 48.0)	12.0 (4.0 to 24.0)	96.0 (48.0 to 144.0)	7.0 (3.0 to 24.0)
Having income from authorities	268 (44.9%)	169 (64.8%)	73 (46.5%)	26 (14.5%)
Having income from work	68 (11.9%)	32 (12.7%)	11 (7.5%)	25 (14.2%)
Migration history				
Country of birth				
Germany	285 (49.2%)	229 (93.5%)	56 (37.8%)	0 (0.0%)
Abroad	294 (50.8%)	16 (6.5%)	92 (62.2%)	186 (100.0%)
Time in Germany, months	360.0 (72.0 to 528.0)	504.0 (396.0 to 612.0)	372.0 (192.0 to 540.0)	24.0 (6.0 to 60.0)
Access to healthcare				
Having health insurance	410 (66.8%)	239 (89.5%)	97 (61.4%)	74 (39.2%)
Visiting a doctor in < 12 months	440 (72.4%)	206 (78.0%)	123 (78.3%)	111 (59.4%)
Visiting a hospital in < 12 months	250 (43.2%)	113 (44.8%)	72 (47.4%)	65 (37.1%)

All variables in this table were included in the latent class analysis.

*PEH*, People experiencing homelessness; *ETHOS*, European typology of homelessness and housing exclusion.

**Table 2:** Psychiatric health status and risk factors of people experiencing homelessness by their most probable class assignment from latent class analysis.

	Total (N = 643) Median (IQR) and number (%)	Class 1 (N = 275) Median (IQR) and number (%)	Class 2 (N = 166) Median (IQR) and number (%)	Class 3 (N = 202) Median (IQR) and number (%)
Psychiatric health status				
EQ-VAS	75.0 (50.0 to 90.0)	75.0 (55.0 to 85.0)	65.0 (40.0 to 80.0)	80.0 (55.0 to 95.0)
Loneliness (UCLA-3 ≥ 6)	253 (42.0%)	122 (45.9%)	79 (51.0%)	52 (28.6%)
Anxiety (GAD-2 ≥ 3)	162 (27.0%)	73 (27.3%)	52 (33.1%)	37 (20.9%)
Moderate and severe depression (PHQ-9 ≥ 14)	94 (48.2%)	33 (12.7%)	41 (26.6%)	20 (11.8%)
Somatic health status				
BMI, kg/m^2^	23.7 (21.5 to 27.4)	23.7 (21.6 to 27.1)	23.4 (21.1 to 28.6)	24.0 (21.6 to 27.2)
SR Liver disease	84 (13.4%)	47 (17.3%)	29 (17.8%)	8 (4.1%)
SR HIV	6 (1.0%)	2 (0.7%)	4 (2.5%)	0 (0.0%)
SR Tuberculosis	8 (1.3%)	3 (11%)	3 (1.8%)	2 (1.0%)
Risk factors				
History of unprotected sex with different partners	115 (21.7%)	44 (18.6%)	36 (25.4%)	35 (23.2%)
History of imprisonment	324 (54.4%)	155 (58.9%)	98 (61.6%)	71 (40.8%)
Current intravenous drug consumption	82 (13.6%)	44 (16.8%)	26 (16.6%)	12 (6.6%)
Time of current intravenous drug consumption, months	102.0 (22.0 to 294.0)	132.0 (21.5 to 300.0)	102.0 (60.0 to 276.0)	20.0 (4.0 to 60.0)
Current receipt of any OAT	66 (11.0%)	36 (13.6%)	27 (17.4%)	3 (1.7%)
Time of current OAT receipt	24.0 (7.0 to 72.0)	24.0 (12.0 to 120.0)	12.0 (7.0 to 24.0)	72.0 (72.0 to 72.0)
Alcohol consumption: > 7 glasses per week				
Never	201 (33.3%)	67 (25.3%)	56 (36.1%)	78 (42.4%)
Occasional	222 (36.8%)	111 (41.9%)	43 (27.7%)	68 (37.0%)
Daily	181 (30.0%)	87 (32.8%)	56 (36.1%)	38 (20.7%)
Time of alcohol consumption, years	15.0 (4.0 to 25.0)	20.0 (4.0 to 26.0)	15.0 (5.0 to 27.0)	10.0 (3.0 to 20.0)
Smoking				
Never	117 (19.3%)	38 (14·2%)	33 (21·0%)	46 (25·3%)
Occasional	32 (5.3%)	14 (5·2%)	4 (2·5%)	14 (7·7%)
Daily	458 (75.5%)	216 (80·6%)	120 (76·4%)	122 (67·0%)
Ever prescription required medication consumption	117 (19.5%)	57 (21·4%)	33 (21·7%)	27 (14·8%)

All variables in this table were included in the latent class analysis, except for somatic health status, time of current intravenous drug consumption, time of current OAT receipt and time of alcohol consumption due to collinearity and significant missingness.

*PEH*, people experiencing homelessness; *EQ5D-VAS*, standardised measure for health-related quality of life; *UCLA-3*, standardised questionnaire for loneliness; *GAD-2*, standardised questionnaire for anxiety disorders; PHQ-9, standardised questionnaire for depression; BMI, body mass index; SR, self-reported; OAT, opioid agonist treatment.

Augmented inverse probability weighting was used to estimate the average causal risk difference in HCV infection risk when comparing a world where all PEH were in prison to one where none had ever been incarcerated. A logit link was used in the propensity score and outcome models. Supplementary Material and Supplementary Fig. 1 provides more detail on this estimation.

## Results

Overall, 675 PEH were recruited, while 32 PEH were excluded due to missing hepatitis and HIV measurements. This resulted in 643 PEH cases from four metropolitan areas in Germany: 200 in Hamburg, 101 in Leipzig, 151 in Frankfurt, and 191 in Munich. Overall, 67% of PEH reported having health insurance, over 70% had seen a doctor, and approximately 40% had been hospitalised in the past year. Supplementary Table 1 presents a comparison of the sociodemographic and medical characteristics between the individuals included and those excluded.

**Fig. 1:**
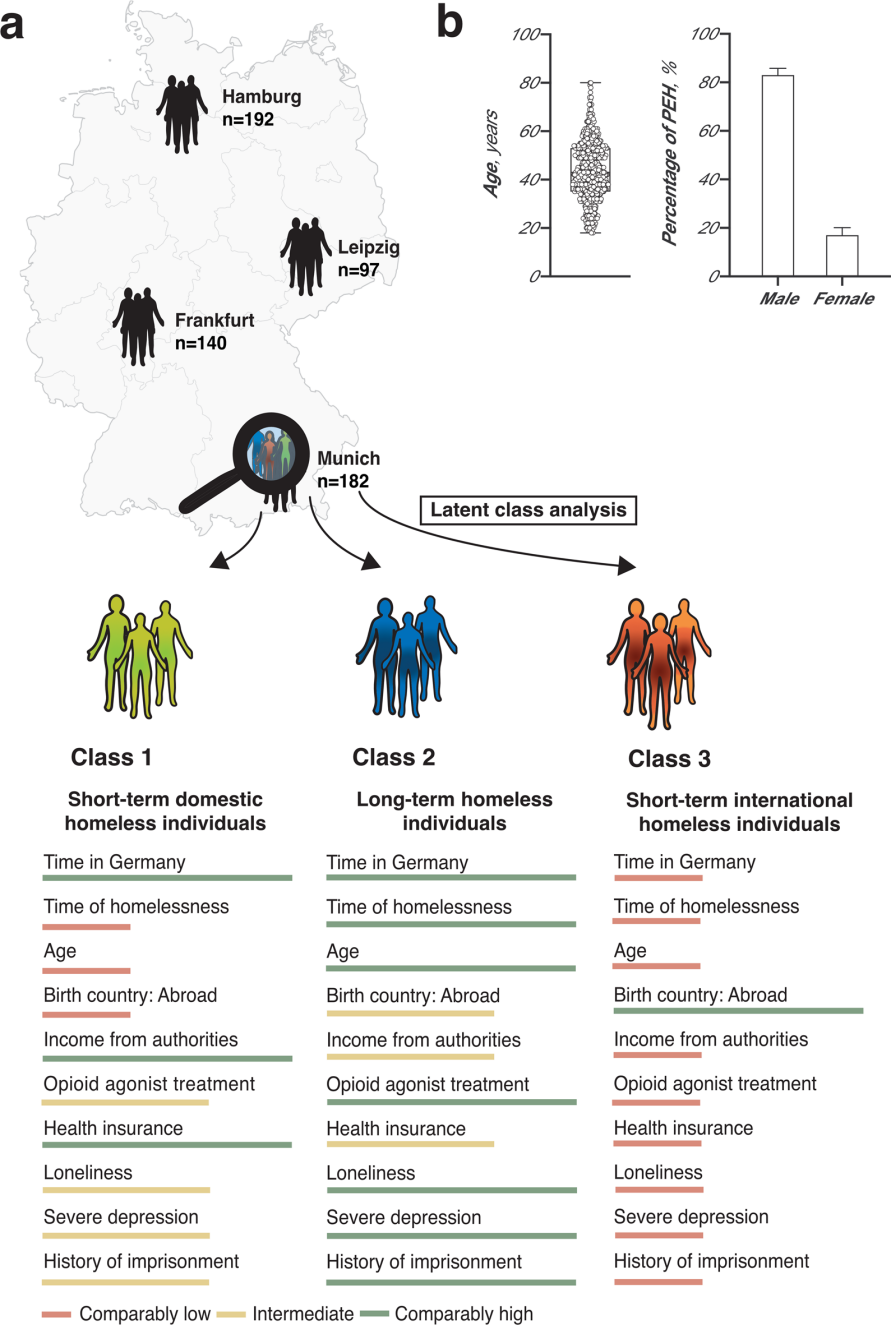
Continuous and categorical variables with the highest degree of separation between classes as obtained from latent class analysis (LCA). Numbers of people experiencing homelessness (PEH) enrolled in each metropolitan area in Germany (**a**). Relative comparisons between variables with the highest degree of separation between the three classes obtained from LCA are illustrated (**a**). Variables were sorted by the root mean squares of the error as obtained from ANOVA. Descriptive statistics of age and sex of PEH enrolled in the study (). 95% confidence intervals are illustrated  (**b**). *Abbreviations*: PEH, people experiencing homelessness; LCA, latent class analysis; ANOVA, analysis of variance.

The mean age of the PEH was 43 years (SD: 12 years), and 17% were female (n = 105). According to the ETHOS classification, 58% of PEH experienced rooflessness (n = 337) and 42% experienced homelessness (n = 249). The sociodemographic and medical characteristics of PEH are shown in Tables 1 and 2. In the LCA, a three-class model provided the best fit to the data, as indicated by the model fit statistics (Supplementary Fig. 2). Consistency of latent class membership for each individual across the 100 imputed datasets is highlighted in Supplementary Fig. 3. Details regarding the latent-class analysis model fit are provided in the Supplementary Material. Time spent homeless, living in Germany, and age were variables that showed the greatest differences across the three latent classes. Additionally, categorical variables such as country of birth, social welfare, participation in opioid agonist treatment (OAT), health insurance status, loneliness as per the UCLA-3, moderate-to-severe depression according to the PHQ-9, and history of imprisonment effectively distinguished the three classes. Figure 1 briefly summarises the continuous and categorical variables that distinguished the classes. A comprehensive summary is provided in Supplementary Fig. 4. Compared to other classes, Class 1 was characterised by a long time living in Germany, a short duration of homelessness, and age in the mid-range of all classes. Class 1 includes most PEH born in Germany with health insurance that received social welfare, with a high prevalence of imprisonment. For participation in OAT programs, prevalence of loneliness according to the UCLA-3 and moderate-to-severe depression according to the PHQ-9, PEH in class 1 were in the mid-range amongst other classes. Class 2 was defined by time living in Germany in the mid-range amongst other classes, a long duration of homelessness, and markedly older age. For numbers being born in Germany, having health insurance, receiving social welfare, and imprisonment history, PEH in class 2 were in the mid-range of all classes. PEH in class 2 showed high participation in OAT programs and the highest prevalence of loneliness according to the UCLA-3 and moderate-to-severe depression according to the PHQ-9. Class 3 was characterised by a very short time living in Germany, a short duration of homelessness, and the youngest age compared to classes 1 and 2. Class 3 was defined by the most PEH born abroad, the fewest PEH with health insurance, the fewest PEH receiving social welfare, and the lowest prevalence of PEH with imprisonment. PEH individuals in class 3 showed low participation in OAT programs, the lowest prevalence of loneliness (according to the UCLA-3, and moderate to severe depression according to the PHQ-9. Hereafter, we refer to these classes as the domestic short-term homelessness class (class 1), long-term homelessness class (class 2), and international short-term homelessness class (class 3).

## Seroprevalence

**Fig. 2:**
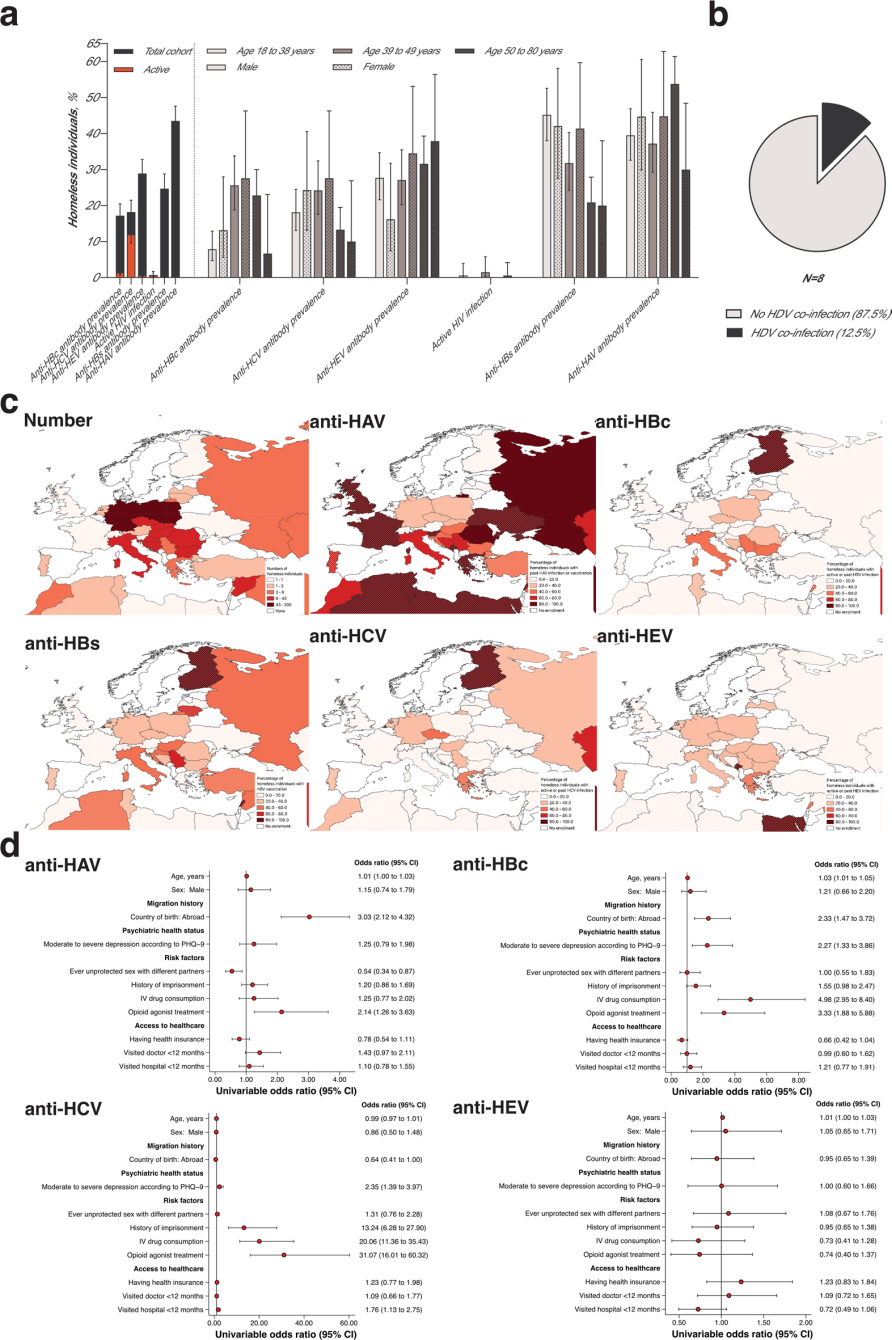
Epidemiological features of viral hepatitis and HIV in People experiencing homelessness (PEH) in Germany. Total and stratified past and active prevalence of viral hepatitis and HIV (**a**). Binomial exact 95% confidence intervals are illustrated. Prevalence of Hepatitis D Virus co-infection among active Hepatitis B Virus-infected individuals (**b**). Prevalence of active or past viral hepatitis according to country of birth in Europe (**c**). Hatching illustrates enrolment of less than 5 PEH. Forrest plots with selected risk factors of active or past infection with viral hepatitis (**d**). *Abbreviations:* PEH, people experiencing homelessness.

The prevalence of viral hepatitis and HIV infection is illustrated in Figs. 2A and 2B. Briefly, the prevalence of anti-HBc IgG antibodies in the cohort was 17% (95%CI: 14-21%), with 1·4% of PEH presenting with HBsAg-positive active HBV infection (95%CI: 0·7-2·7%) with a median viral load of 58 IU/ml [IQR: 15–502 IU/ml]). One individual was diagnosed with HBV/HDV co-infection (12·5% [95%CI: 0·3–52·7%]) The prevalence of anti-HCV antibodies was 18% (95%CI: 15–22%), with a total of 12·0% of PEH presenting with active HCV infection (95%CI: 9·6-14·9%). Here, virus loads ranged between 47 and 2 × 10^7^ IU/ml (median: 4·6 × 10^5^ IU/ml [95%CI: 1·6 × 10^5^ – 2·1 × 10^6^ IU/ml]). The prevalence of anti-HEV IgG antibodies was 29% (95%CI: 25–33%), with 0·7% of PEH presenting with active HEV infections (95%CI: 0·3–1·8%). HEV RNA loads ranged between 28 and 173 IU/ml (median: 131 IU/ml [95%CI: 58 – 173 IU/ml]). No cases of active ratHEV infections were observed. In total, four HIV infections (0·7% [95%CI: 0·2–1·7%]) were diagnosed in the cohort. Here, virus loads were between < LOD and 9·3 × 10^3^ copies/ml (median: 299 copies/ml [IQR: 21-4·9 × 10^3^ copies/ml]). All PEH, except one, were aware of their HIV infection status. In this patient, viral loads were the highest, with 9·3 × 10^3^ copies/ml. The prevalence of anti-HAV IgG antibodies indicating vaccination or past infection in patients with PEH was 44% (95%CI: 39–48%). Among the 485 anti-HBc antibody-negative PEH, the prevalence of anti-HBs antibodies was 25% (95%CI: 21–29%), indicating prior HBV vaccination. Because some measurements were missing (refused consent or no blood sample available; n = 57, n = 59, n = 59, n = 38, n = 57 for anti-HBcAg, anti-HCV, anti-HEV IgG, anti-HIV, and anti-HAV, respectively), we compared the characteristics of PEH with and without available measurements. No substantial differences in the sociodemographic characteristics were observed (Supplementary Tables 4–7).For baseline clinical characteristics of PEH with hepatitis and HIV infection see Supplementary Tables 2–3.

Among the PEH with active or past hepatitis virus infection, 35% with anti-HBc antibodies (n = 34), 50% with anti-HCV antibodies (n = 52), and 10% with anti-HEV antibodies (n = 17) were previously diagnosed with liver disease. Among the PEH with active HBV infection, 50% knew about their diagnosis in advance (95%CI: 11–89), and among the PEH with active HCV infection, 44% knew about their diagnosis in advance (95%CI: 33–57). Co-infection with HBV, HCV, and HIV was examined using Venn diagrams (Supplementary Fig. 5). 7% were anti-HBc and anti-HCV antibody positive (n = 39), 0·2% were anti-HCV and HIV positive (n = 1), and 0·2% were anti-HBc and anti-HCV antibody and HIV positive (n = 1).

Next, acute and past hepatitis and HIV prevalence according to the country of birth were investigated (n = 613; Fig. 2C and Supplementary Figs. 6 to 11). In brief, many PEHs were born in Germany and south-eastern European countries (Fig. 2C). A high prevalence of anti-HAV antibodies was found in PEH from south-eastern European countries, most notably in the Balkan region. The highest prevalence of anti-HBc antibodies was found in PEH from Poland, Italy, and countries in the Balkan region, whereas the highest prevalence of anti-HCV antibodies was observed in PEH from Germany and countries in the Balkan region.

### Specific risk factors

To better understand the primary risk factors for viral hepatitis and HIV infection in PEH, we examined the association of various risk factors with active and past hepatitis and HIV infection status. Odds ratios (ORs) and 95% confidence intervals (95%CIs) were calculated using an univariable logistic regression analysis (Fig. 2D). For HAV, homeless individuals born abroad had 3·03 times the odds of past HAV infection or vaccination than individuals born in Germany (95%CI: 2·12–4·32, p < 0·001). For HBV, homeless individuals born abroad had 2·33 times the odds of active or past HBV infection that individuals born in Germany had (95%CI: 1·47–3·72, p < 0·001), individuals with moderate to severe depression, had 2·27 times the odds of active or past HBV infection (95%CI: 1·33–3·86, p = 0·003) that individuals without had. Individuals with active intravenous drug consumption had 4.98 times the odds of active or past HBV infection (95%CI: 2·95–8·40, p < 0·001) that individuals without had, and individuals participating in an OAT program had 3.33 times the odds of active or past HBV infection (95%CI: 1·88–5·88, p < 0·001) that those who did not have. For HCV, homeless individuals with a history of imprisonment had 13.24 times the odds of active or past HCV infection (95%CI: 6·28–27·90, p < 0·001) that individuals without had, individuals with active i.v. drug consumption had 20·06 times the odds of active or past HCV infection (95%CI: 11·36–35·43, p < 0·001) that individuals without had, and people with OAT had 31·07 times the odds of active or past HCV infection that people without had (95%CI: 16·01–60·32, p < 0·001). Further details are provided in Supplementary Figs. 12–17. No evidence of effect modification was found, suggesting that a history of imprisonment affects PEH with and without active intravenous drug consumption differently (p = 0·96). If all PEH were imprisoned, the probability of HCV infection was 25·4% (95%CI: 20·2–30·6), and if all PEH were not 6·1% (95%CI: 1·4–10·8). Controlling for confounding using augmented inverse probability weighting, the average causal risk difference in HCV infection was 19·3% (95%CI: 12·5–26·1), comparing a hypothetical world where all PEH are imprisoned with a hypothetical world where all PEH are not. Further details are provided in Supplementary Material and Supplementary Fig. 1.

### Composite risk of subgroups of homeless individuals

Table 3 summarises the association between PEH classes and the risk of hepatitis infection. For active or past HBV infection, a prevalence of active or past HBV infection of 12·0% (95%CI: 8·0-15·9%) in domestic short-term homeless individuals, 23·8% (95%CI: 17·0-30·6%; OR: 2·30 [95%CI: 1·36-3·91], p = 0·002) in long-term homeless individuals, and 19·3% (95%CI: 13·5-25·2%; OR: 1·76 [95%CI: 1·04-2·99], p = 0·04) in international short-term homeless individuals was observed. For active or past HCV infection, a prevalence of active or past HCV infection of 20·1% (95%CI: 15·2-25·0%) in domestic short-term homeless individuals, 25·3% (95%CI: 18·4-32·3%; OR: 1·35 [95%CI: 0·84-2·18], p = 0·22) in long-term homeless individuals, and 9·1% (95%CI: 4·9-13·4%; OR: 0·40 [95%CI: 0·22-0·73], p = 0·003) in international short-term homeless individuals was observed. For past HAV infection or vaccination, a prevalence of past HAV infection or vaccination of 33·6% (95%CI: 27·8-39·3%) in domestic short-term homeless individuals, 48·3% (95%CI: 40·4-56·3%; OR: 1·85 [95%CI: 1·23-2·79], p = 0·003) in long-term homeless individuals, and 54·0% (95%CI: 46·6-61·3%; OR: 2·32 [95%CI: 1·57-3·43], p < 0·001) in international short-term homeless individuals was observed. For HBV vaccination, a prevalence of HBV vaccination of 28·1% (95%CI: 22·2-33·9%) in domestic short-term homeless individuals, 18·3% (95%CI: 11·1-25·3%; OR: 0·57 [95%CI: 0·33-1·00], p = 0·05) in long-term homeless individuals, and 24·6% (95%CI: 17·6-31·7%; OR: 0·84 [95% CI: 0·52-1·35], p = 0·47) in international short-term homeless individuals was observed (Table 3).

**Table 3:** Assessing the odds of active or past infection with viral hepatitis or vaccination in people experiencing homelessness by most probable class assignment.

	Prevalence of viral hepatitis per class		
	Viral Hepatitis prevalence (95% CI)	Odds ratio (95% CI)	P-value
Past infection or vaccination with hepatitis A virus (anti-HAV antibodies)^1^			
Overall cohort	43.5 (39.5 to 47.6)		
Class 1	33.6 (27.8 to 39.3)	Reference	
Class 2	48.3 (40.4 to 56.3)	1.85 (1.23 to 2.79)	0.003
Class 3	54.0 (46.6 to 61.3)	2.32 (1.57 to 3.43)	< 0.001
Active or past infection with hepatitis B virus (anti-HBc antibodies)^1^			
Overall cohort	17.2 (14.4 to 20.5)		
Class 1	12.0 (8.0 to 15.9)	Reference	
Class 2	23.8 (17.0 to 30.6)	2.30 (1.36 to 3.91)	0.002
Class 3	19.3 (13.5 to 25.2)	1.76 (1.04 to 2.99)	0.04
Vaccination for Hepatitis B (anti-HBs antibodies)^2^			
Overall cohort	24.7 (21.1 to 28.8)		
Class 1	28.1 (22.2 to 33.9)	Reference	
Class 2	18.3 (11.1 to 25.3)	0.57 (0.33 to 1.00)	0.05
Class 3	24.6 (17.6 to 31.7)	0.84 (0.52 to 1.35)	0.47
Active or past infection with hepatitis C virus (anti-HCV antibodies)^3^			
Overall cohort	18.2 (15.2 to 21.5)		
Class 1	20.1 (15.2 to 25.0)	Reference	
Class 2	25.3 (18.4 to 32.3)	1.35 (0.84 to 2.18)	0.22
Class 3	9.1 (4.9 to 13.4)	0.40 (0.22 to 0.73)	0.003
Active or past infection with hepatitis E virus (anti-HEV antibodies)^3^			
Overall cohort	28.9 (25.3 to 32.8)		
Class 1	28.8 (23.2 to 34.4)	Reference	
Class 2	32.5 (25.2 to 39.8)	0.91 (0.59 to 1.42)	0.69
Class 3	28v9 (19.4 to 32.4)	0.86 (0.56 to 1.33)	0.49

Homeless individuals in Germany can be categorised into three main groups: domestic short-term homeless individuals (class 1), long-term homeless individuals (class 2), and international short-term homeless individuals (class 3). Logistic regression was used to obtain odds ratios and 95% confidence intervals.

*PEH*, people experiencing homelessness.

^1^Data is available for 586 homeless individuals. ^2^Data is available for 485 homeless individuals without acute or past HBV infection. ^3^Data is available for 584 homeless individuals.

## Discussion

WHO aims to reduce the burden of viral hepatitis substantially by 2030, but current trajectories suggest this aim will be missed^3^. From a public health perspective, PEH may be a reservoir for infectious diseases^4^. At the time of data acquisition, 607.000 individuals experienced homelessness in Germany. This estimate includes individuals experiencing hidden homelessness, temporarily living with friends and family, as well as those seeking help in institutions or sleeping rough. Data on the prevalence of HIV and viral hepatitis, as well as data on vaccine coverage within subgroups, may help identify individuals benefiting from targeted interventions. We conducted a nationwide multicentre cross-sectional study, collecting clinical and laboratory data from 643 PEH in four regions of Germany.

High engagement of PEH with the healthcare system encourages optimism for successful future interventions, as it may enable structured screening efforts and the transition of PEH into long-term treatment within regular German healthcare services. The sociodemographic characteristics of PEH were highly diverse, consistent with the sample characteristics reported in other homeless cohorts in Germany and Europe. High rates of male participants may be caused by the higher frequency of hidden homelessness in women^13^. Like other sociodemographics, the migration patterns of PEH may be influenced by the unique geographical, historical, and political conditions of the countries. LCA was used to identify three different classes in the cohort of PEH: the domestic short-term homelessness class, the long-term homelessness class, and the international short-term homelessness class. These subgroups allow an understanding of the diversity of the cohort and reduce data complexity.

The prevalence of anti-HEV IgG was 29% in our cohort, with four individuals testing positive for HEV RNA. A similar prevalence of active HEV and anti-HEV antibodies was in German blood donors, showing no increased risk of HEV infection in PEH compared to the general population^14^. Moreover, no case of rat HEV was detected^15^. Anti-HAV IgG prevalence was 44%, which was slightly lower than that reported for the general German population in 2013 (48% [95%CI: 47·0–50·2%])^16^. Universal recommendations for HAV vaccination in PEH were made in the USA after studies showed that PEH accounted for half of all outbreak-associated cases^17^. Overall, the data show the potential of healthcare interventions to increase hepatitis A vaccination coverage among PEH. Interestingly, we observed a three-fold increase in the odds of anti-HAV antibody positivity in PEH of non-German origin compared to that in PEH of German origin, which is presumably driven by the acquired immunity of individuals migrating from endemic regions^18^. Among individuals without a history of HBV infection, the prevalence of anti-HBs antibodies is only 25%, indicating a low prevalence of vaccine-induced immunity. These data align with previous monocentric analysis of anti-HBs antibody prevalence performed in PEH in 2020 in Hamburg, Germany, and the prevalence reported in the general German population (20–25%)^9,16^. In Germany, vaccination against HBV is universally recommended in children, and an extended vaccination scheme may be applied in patients with risk factors, such as immunosuppression, intravenous drug use, or residence in a psychiatric facility^6^. Revisiting the recommendations of the Standing Committee on Vaccination (STIKO) and adopting the USA’s example of recognizing homelessness as an independent indication for HAV and HBV vaccination can help achieve the WHO goal. International intervention studies have indicated that nurse-led vaccination programs can result in HAV and HBV immunity in up to 75% of previously unvaccinated PEH, thereby demonstrating the great potential to enhance vaccination coverage among PEH in Germany^19^.

The prevalence of anti-HBcAg was 17%, which was more than three times higher than that in the general German population (5%)^20^. This observation aligns with previous findings from small and monocentric German studies^9,10^. The odds of anti-HBc antibody positivity were increased in individuals born abroad compared with those born in Germany, and the observed prevalence in the countries of origin corresponded to known high-risk regions^21^. Increased odds of anti-HBc antibody positivity were also observed in individuals who reported previous imprisonment, intravenous drug consumption, or participation in OAT programs. Overall, these population-based findings confirm risk factors for active or past HBV infection among PEH^22^. Likewise, the proportion of HDV-coinfected individuals among those with active HBV aligns with the published literature in the general population^23^. Concerning HIV, in total, four individuals (0·7%) were diagnosed with HIV, and all but one were aware of their infection. The overall prevalence was lower than that of a monocentric study performed in Berlin in 2021, where 2·8% (95%CI: 1·0–5·6%) of the total cohort tested positive for HIV^10^. Selection bias may explain the divergent HIV prevalence among the studies^24^. In both studies, the prevalence of HIV in PEH was higher than that in the general German population. Fortunately, intervention studies show high acceptance of HIV testing programs in PEH, which calls for structured testing in subgroups of PEH^25^.

The overall prevalence of anti-HCV was 18%, with 66% (n = 70) found to have active HCV infections. This corresponds to a 10-to-100-fold increase in the prevalence of anti-HCV antibodies compared to the range of 0·2 to 1·9% in the German general population. With an estimated 607,000 homeless individuals in Germany, our data would indicate that 72,840 homeless individuals in Germany (95%CI: 58,272–90,443) have active HCV infection with immediate treatment indications^8^. A high prevalence of anti-HCV antibodies and active HCV infection in PEH has also been reported in a monocentric cross-sectional study of 216 PEH in Berlin, Germany, and a monocentric cross-sectional study of 491 individuals from vulnerable populations (including PEH) from London, UK^10,26^. In the literature, the prevalence of anti-HCV antibodies is strongly dependent on the risk behaviour of the studied population, reaching a prevalence of more than 60% among people injecting drugs^27^. Consistent with this, increased odds of anti-HCV antibody positivity were observed in individuals reporting current intravenous drug use or participating in the OAT, indicating former drug use. Interestingly, the odds of anti-HBV and anti-HCV antibody positivity increased in individuals with signs of moderate to severe depression. Explanations for this bidirectional relationship include stigma, awareness of the chronic illness, and an increased prevalence of risky behaviour in individuals with mental health problems^28^.

Increased odds of anti-HBc and anti-HCV antibody positivity were observed in individuals who had reported former imprisonment. Here, more than 90% of individuals with active or past HCV infection reported incarceration, emphasizing the drastic increase in the risk of HCV infection associated with previous imprisonment. In Australia, studies have identified prison-based interventions as the primary driver of HCV elimination^29^. Here, the HCV risk attributable to imprisonment was estimated to be 19%. Despite knowledge about the high prevalence and transmission rates of HCV in prisons, no universal practice of testing and treating viral hepatitis in German prisons has been established yet^30^. Here, the findings urgently call for screening and treatment of HCV and raise critical questions regarding the ethical and political responsibilities of ensuring equitable healthcare access for this vulnerable population.

Based on LCA, HCV screening should be offered to domestic, short-term, and long-term homeless individuals. Targeted interventions should aim to refer to domestic short- and long-term PEH with suspected HCV treatment indications to regular care providers, as they are likely to have health insurance. Importantly, linkage to care was shown to be highly effective in vulnerable populations outside of institutions, achieving a sustained virological response in more than 80% of participants^31^. The European Center for Disease Prevention and Control highlights the use of community-based programs involving peers and measures to increase the trust between vulnerable cohorts and care providers^32^.

### Strengths and limitations

This is the first nationwide, multicentre, cross-sectional study of all viral hepatitis entities in PEH. Participants were recruited from various aid services to represent the PEH community in Germany. However, assessing the representativeness of this dataset remains challenging as the response rates are missing. Despite the availability of translation services, language barriers among immigrants and not reaching individuals experiencing hidden homelessness may have introduced selection bias. This study included a comprehensive assessment of clinical and laboratory parameters and risk profiles. However, not all risk factors were included, as data on sexual practices were not evaluated.

## Conclusion

This national multicentre cross-sectional study provides updated data on sociodemographic characteristics, infection status for viral hepatitis and HIV, and vaccine coverage in PEH. Considering the high prevalence of active infections (HCV, HBV, HIV) and low prevalence of immune protection (HAV and HBV) in the cohort, our study calls for national screening, treatment, and prevention programs for PEH. Establishing homelessness as an independent indication for vaccination should be considered, and implementing screening and treatment of HCV in prisons is urgently needed.

## Supplementary Information

Below is the link to the electronic supplementary material.

Supplementary Material 1

## Data Availability

Data collected in this study, including de-identified individual participant data and a data dictionary defining each variable of the set, will be made available to others upon request. Please contact Dr Fabian Heinrich at fa.heinrich@uke.de.

## Abbreviations

AIC: Akaike information criteria
BIC: Bayesian information criteria
BMI: Body mass index
CE-IVD: European in-vitro diagnostic devices directive
EQ5D-VAS: Standardized measure for health-related quality of life
ETHOS: European typology of homelessness and housing exclusion
GAD-2: Standardized questionnaire for anxiety disorders
HAV: Hepatitis A Virus
HBV: Hepatits B Virus
HCV: Hepatitis C Virus
HDV: Hepatitis D Virus
HEV: Hepatitis E Virus
HIV: Human immunodeficiency Virus
LCA: Latent class analysis
LOD: Limit of detection
MCH: Mean corpuscular hemoglobin
MCV: Mean corpuscular volume
MSM: Men who have sex with men
NAPSHI: National survey on psychiatric and somatic health of homeless individuals
OAT: Opioid agonist treatment
PEH: People experiencing homelessness
PHQ-9: Standardized questionnaire for depression
SR: Self-reported
UCLA-3: Standardized questionnaire for loneliness
USA: United States of America
WHO: World Health Organization

## References

[1] WHO. Elimination of hepatitis by 2030 Geneva [Available from: https://www.who.int/health-topics/hepatitis/elimination-of-hepatitis-by-2030#tab=tab_1.

[2] Gesundheitsministerium. Strategie zur Eindämmung von HIV, Hepatitis B und C und anderen sexuell übertragbaren Infektionen Germany [Available from: https://www.bundesgesundheitsministerium.de/fileadmin/Dateien/5_Publikationen/Praevention/Broschueren/Strategie_BIS_2030_HIV_HEP_STI.pdf.

[3] WHO. Guidance for country validation of viral hepatitis elimination and path to elimination 2023 [Available from: https://www.who.int/publications/i/item/9789240078635. 10.1016/S2468-1253(21)00267-3 34384530

[4] Badiaga, S., Raoult, D. & Brouqui, P. Preventing and controlling emerging and reemerging transmissible diseases in the homeless. *Emerg Infect Dis.* **14** (9), 1353–1359 (2008). 18760000 10.3201/eid1409.082042 PMC2603102

[5] Bertram, F. et al. The mental and physical health of the homeless. *Dtsch Arztebl International.* **119**(50), 861–868 (2022). 10.3238/arztebl.m2022.0357 PMC9989961 36382585

[6] RKI. Recommendation by the Standing Committee on Vaccination (STIKO) at the Robert Koch Institute. *Epidemiologisches Bulletin* (2023).

[7] Omerov, P., Craftman, Å. G., Mattsson, E. & Klarare, A. Homeless persons’ experiences of health- and social care: A systematic integrative review. *Health Soc Care Community.* **28** (1), 1–11 (2020). 31524327 10.1111/hsc.12857

[8] Bundesarbeitsgemeinschaft Wohnungslosenhilfe e. V. Mindestens 607.000 Menschen in Deutschland wohnungslos 2023 [Available from: https://www.bagw.de/fileadmin/bagw/media/Doc/PRM/PRM_PM_BAG_W_Pressemappe_Hochrechnung_Zahl_der_wohnungslosen_Menschen.pdf.

[9] Heinrich, F. et al. The prevalence and determinants of viral hepatitis among homeless individuals in hamburg. *Dtsch Arztebl Int.* **119**(1–2), 8–9 (2022). 37255009 10.3238/arztebl.m2022.0003 PMC9002301

[10] Steffen, G. et al. Prävalenz von sexuell und durch Blut übertragenen Infektionen und Tuberkulose bei Menschen in Wohnungs­losigkeit in Berlin – Erste Ergebnisse der Pilotstudie POINT. *Epidemiologisches Bulletin.* **13**, 25–32 (2022).

[11] Cordes, A. K. et al. Risk of transfusion-transmitted hepatitis E virus infection from pool-tested platelets and plasma. *J. Hepatol.* **76**(1), 46–52 (2022). 34461207 10.1016/j.jhep.2021.08.018

[12] Sinha, P., Calfee, C. S. & Delucchi, K. L. Practitioner’s guide to latent class analysis: methodological considerations and common pitfalls. *Crit Care Med.* **49**(1), e63–e79 (2021). 33165028 10.1097/CCM.0000000000004710 PMC7746621

[13] Tinland, A. et al. Mortality in homeless people enrolled in the French housing first randomized controlled trial: A secondary outcome analysis of predictors and causes of death. *BMC Public Health* **21**(1), 1294 (2021). 34215235 10.1186/s12889-021-11310-w PMC8254224

[14] Hartl, J. et al. Hepatitis E seroprevalence in Europe: A meta-analysis. *Viruses.* **8**(8), 211 (2016). 27509518 10.3390/v8080211 PMC4997573

[15] Rivero-Juarez, A. et al. Orthohepevirus C infection as an emerging cause of acute hepatitis in Spain: First report in Europe. *J Hepatol.* **77**(2), 326–331 (2022). 35167911 10.1016/j.jhep.2022.01.028

[16] Poethko-Muller, C. et al. Epidemiology of hepatitis A, B, and C among adults in Germany: results of the German health interview and examination survey for adults (DEGS1). *Bundesgesundheitsblatt Gesundheitsforschung Gesundheitsschutz* **56**(5–6), 707–715 (2013). 23703489 10.1007/s00103-013-1673-x

[17] Nelson, N. P. et al. Prevention of hepatitis A virus infection in the united states: Recommendations of the advisory committee on immunization practices, 2020. *MMWR Recomm Rep.* **69** (5), 1–38 (2020). 32614811 10.15585/mmwr.rr6905a1 PMC8631741

[18] European Centre for Disease Prevention and Control. Hepatitis A. Stockholm: ECDC: 2022 [Available from: https://www.ecdc.europa.eu/sites/default/files/documents/HEPA_AER_2022_Report.pdf.

[19] Nyamathi, A. et al. Nursing case management, peer coaching, and hepatitis a and B vaccine completion among homeless men recently released on parole: Randomized clinical trial. *Nurs Res.* **64**(3), 177–189 (2015). 25932697 10.1097/NNR.0000000000000083 PMC4418035

[20] Brodzinski, A. et al. Hepatitis B virus infection and vaccine-induced immunity: the role of sociodemographic determinants: Results of the study “German health interview and examination survey for adults” (DEGS1, 2008–2011). *Bundesgesundheitsblatt Gesundheitsforschung Gesundheitsschutz* **65**(2), 159–169 (2022). 34958395 10.1007/s00103-021-03473-z PMC8813829

[21] European Centre for Disease Prevention and Control. Hepatitis B. Stockholm: ECDC: 2022 [Available from: https://www.ecdc.europa.eu/sites/default/files/documents/AER%20HEPB%202022_0.pdf.

[22] Jeng, W. J., Papatheodoridis, G. V. & Lok, A. S. F. Hepatitis B. *Lancet* **401**(10381), 1039–1052 (2023). 36774930 10.1016/S0140-6736(22)01468-4

[23] Chen, H. Y. et al. Prevalence and burden of hepatitis D virus infection in the global population: a systematic review and meta-analysis. *Gut* **68**(3), 512–521 (2019). 30228220 10.1136/gutjnl-2018-316601

[24] Beijer, U., Wolf, A. & Fazel, S. Prevalence of tuberculosis, hepatitis C virus, and HIV in homeless people: a systematic review and meta-analysis. *Lancet Infect Dis.* **12**(11), 859–870 (2012). 22914343 10.1016/S1473-3099(12)70177-9 PMC3494003

[25] Pichon, L. C. et al. A pilot outreach HIV testing project among homeless adults. *Front Public Health.* **9**, 703659 (2021). 34395372 10.3389/fpubh.2021.703659 PMC8356944

[26] Aisyah, D. N. et al. Hepatitis C among vulnerable populations: A seroprevalence study of homeless, people who inject drugs and prisoners in London. *J. Viral. Hepat.* **25**(11), 1260–1269 (2018). 29851232 10.1111/jvh.12936

[27] Sperle, I. et al. Prevalence of hepatitis B, C, and D in Germany: Results from a scoping review. *Front Public Health.* **8**, 424 (2020). 33014960 10.3389/fpubh.2020.00424 PMC7493659

[28] Fu, H. et al. Causal associations between chronic viral hepatitis and psychiatric disorders: A Mendelian randomization study. *Front Psychiatry.* **15**, 1359080 (2024). 38881548 10.3389/fpsyt.2024.1359080 PMC11176532

[29] Stone, J. et al. Prison-based interventions are key to achieving HCV elimination among people who inject drugs in New South Wales, Australia: A modelling study. *Liver Int.* **43** (3), 569–579 (2023). 36305315 10.1111/liv.15469 PMC10308445

[30] Dichtl, A. et al. Hepatitis C in prisons: Treatment and barriers to the elimination goals of the United Nations. *Bundesgesundheitsblatt Gesundheitsforschung Gesundheitsschutz* **67** (1), 36–44 (2024). 38155308 10.1007/s00103-023-03808-y PMC10776704

[31] Mourad, A. et al. A Novel Multisite Model to Facilitate Hepatitis C Virus Elimination in People Experiencing Homelessness. JHEP Reports. 2024. 10.1016/j.jhepr.2024.101183 PMC11546132 39524209

[32] ECDC. European Centre for Disease Prevention and Control. Models of good practice for community-based testing, linkage to care and adherence to treatment for hepatitis B and C, HIV, and tuberculosis and forhealth promotion interventions to prevent infections among people who inject drugs. 2022 [Available from: https://www.ecdc.europa.eu/sites/default/files/documents/Good-practice-for-community-based-testing.pdf.

