# Supplementary material for: Prevalence and risk factors of viral hepatitis and HIV among people experiencing homelessness in Germany based on a nationwide study
Source: Sci Rep. 2025 Sep 17;15:32571. doi: 10.1038/s41598-025-18552-3 (PMC12443954; doi:10.1038/s41598-025-18552-3)
Supplement: Supplementary file 1 — Supplementary Material 1 [file 41598_2025_18552_MOESM1_ESM.docx]

**Prevalence and Risk Factors of Viral Hepatitis and HIV Among People Experiencing Homelessness in Germany Based on a Nationwide Study**

Fabian Heinrich^1,2,3*#^, Tsz Lun Ernest Wong^4*^, Wiebke Graf^1^, Katharina Dost^1^, Anna Brennecke^1^, Veronika Kowalski^1^, Victoria van Rüth^1,5^, Stefanie Iwersen-Bergmann^1^, André Hajek^6^, Hans-Helmut König^6^, Thomas Renné^7,8,9^, Thomas Theo Brehm^10,11,12^, Susanne Pfefferle^13^, Julian Schulze zur Wiesch^10,11,12^, Maura Dandri^10,12^, Martin Aepfelbacher^13^, Klaus Püschel^1^, Benjamin Ondruschka^1^, Marc Lütgehetmann^12,13*#^, Franziska Stallbaum^*^

**Supplementary Material**

**Table of content**

- **Supplementary Table 1.** Baseline characteristics of PEH enrolled and not enrolled in the analytic sample are illustrated.
- **Supplementary Table 2.** Clinical characteristics of PEH with active hepatitis B infection, active hepatitis C infection, active hepatitis D infection, and active hepatitis E infection.
- **Supplementary Table 3.** Clinical characteristics of PEH with active HIV infection.
- **Supplementary Table 4.** Baseline characteristics of PEH with available and unavailable hepatitis A and B status are illustrated.
- **Supplementary Table 5.** Baseline characteristics in PEH with available and unavailable hepatitis C status are illustrated.
- **Supplementary Table 6.** Baseline characteristics in PEH with available and unavailable hepatitis E status are illustrated.
- **Supplementary Table 7.** Baseline characteristics in PEH with available and unavailable HIV status are illustrated.
- **Supplementary Figure 1.** Directed acyclic graph (drawn using DAGitty(1)) on the causal relationship between imprisonment and hepatitis C infection.
- **Supplementary Figure 2.** Latent class fit statistics by the number of latent classes.
- **Supplementary Figure 3.** Distribution of class assignment frequencies across 100 imputation datasets by latent class.
- **Supplementary Figure 4.** The standardised mean of continuous variables by class (A) and frequencies of categorical variables by class (B and C) are illustrated with decreasing degree of separation between classes. Variables were sorted by the mean squares of error from ANOVA.
- **Supplementary Figure 5.** Venn diagram for overlap between parenteral transmitted active HIV and active or past hepatitis infections. Non-missing percentages are shown for cases with complete measurements for all infections illustrated.
- **Supplementary Figure 6.** Numbers of PEH in the German homeless population according to the country of birth.
- **Supplementary Figure 7.** Percentage of PEH diagnosed with past HAV infection and/or vaccination according to the country of birth. White hatching illustrates enrolment numbers below 5.
- **Supplementary Figure 8.** Percentage of PEH diagnosed with active or past HBV infection in the German homeless population according to the country of birth. White hatching illustrates enrolment numbers below 5.
- **Supplementary Figure 9.** Percentage of PEH diagnosed with active or past HCV infection in the German homeless population according to the country of birth. White hatching illustrates enrolment numbers below 5.
- **Supplementary Figure 10.** Percentage of PEH diagnosed with active or past HEV infection in the German homeless population according to the country of birth. White hatching illustrates enrolment numbers below 5.
- **Supplementary Figure 11.** Percentage of PEH diagnosed with HBV vaccination among individuals without active or past HBV infection in the German homeless population according to the country of birth. White hatching illustrates enrolment numbers below 5.
- **Supplementary Figure 12.** Odds ratios and 95% confidence intervals with past HAV infection or vaccination as the model-dependent variable are illustrated.
- **Supplementary Figure 13.** Odds ratios and 95% confidence intervals with active or past HBV infection as the model-dependent variable are illustrated.
- **Supplementary Figure 14.** Odds ratios and 95% confidence intervals with HBV vaccination in homeless individuals without active or past HBV infection as the model-dependent variable are illustrated.
- **Supplementary Figure 15.** Odds ratios and 95% confidence intervals with active or past HCV infection as the model-dependent variable are illustrated.
- **Supplementary Figure 16.** Odds ratios and 95% confidence intervals with active or past HEV infection as the model-dependent variable are illustrated.
- **Supplementary Figure 17.** Odds ratios and 95% confidence intervals with active HIV infection as the model-dependent variable are illustrated. Firth bias correction was applied. Self-reported HIV was excluded due to strong collinearity.
- **Supplementary Material.** Key steps in latent class analysis.
- **Supplementary Material.** Modelling results for the LCA.
- **Supplementary Material.** Estimating the causal effect of imprisonment on HCV infection rates

**Supplementary Table 1.** Baseline characteristics of PEH enrolled and not enrolled in the analytic sample are illustrated.

|  | **Patients not enroled in the analytic sample**  N=32 | **Patients enroled in the analytic sample**  N=643 | **Total**  N=675 |
| --- | --- | --- | --- |
| Age, years | 42·5 (35·0 to 51·0) | 43·0 (35·0 to 53·0) | 43·0 (35·0 to 52·0) |
| Sex |  |  |  |
| Female | 13 (43·3%) | 105 (17·0%) | 118 (18·2%) |
| Male | 17 (56·7%) | 514 (83·0%) | 531 (81·8%) |
| Educational attainment |  |  |  |
| No degree | 6 (20·0%) | 107 (18·0%) | 113 (18·1%) |
| School education | 11 (36·7%) | 276 (46·5%) | 287 (46·0%) |
| Higher education | 13 (43·3%) | 211 (35·5%) | 224 (35·9%) |
| Marital status |  |  |  |
| Single | 19 (63·3%) | 401 (67·2%) | 420 (67·0%) |
| Married | 5 (16·7%) | 69 (11·6%) | 74 (11·8%) |
| Divorced/widowed | 6 (20·0%) | 127 (21·3%) | 133 (21·2%) |
| **Characteristics of homelessness** | | | |
| City of enrolment |  |  |  |
| Hamburg | 13 (40·6%) | 200 (31·1%) | 213 (31·6%) |
| Frankfurt | 3 (9·4%) | 151 (23·5%) | 154 (22·8%) |
| Leipzig | 7 (21·9%) | 101 (15·7%) | 108 (16·0%) |
| Munich | 9 (28·1%) | 191 (29·7%) | 200 (29·6%) |
| ETHOS classification |  |  |  |
| Roofless | 16 (55·2%) | 337 (57·5%) | 353 (57·4%) |
| Homeless | 13 (44·8%) | 249 (42·5%) | 262 (42·6%) |
| Time of homelessness, months | 24·0 (5·0 to 48·0) | 1·.0 (5·0 to 48·0) | 18.0 (5·0 to 48·0) |
| Having income from authorities | 19 (63·3%) | 268 (44·9%) | 287 (45·8%) |
| Having income from work | 5 (17·9%) | 68 (11·9%) | 73 (12·1%) |
| **Migration history** | | | |
| Country of birth |  |  |  |
| Germany | 16 (55·2%) | 285 (49·2%) | 301 (49·5%) |
| Abroad | 13 (44·8%) | 294 (50·8%) | 307 (50·5%) |
| Time in Germany, months | 444·0 (240·0 to 588·0) | 360·0 (72·0 to 528·0) | 360·0 (72·0 to 540·0) |
| **Psychiatric health status** | | | |
| EQ-VAS | 70·0 (50·0 to 80·0) | 75·0 (50·0 to 90·0) | 75·0 (50·0 to 90·0) |
| Loneliness (UCLA-3 ≥6) | 11 (36·7%) | 253 (42·0%) | 264 (41·7%) |
| Anxiety (GAD-2 ≥ 3) | 9 (30·0%) | 162 (27·0%) | 171 (27·1%) |
| Moderate to severe depression (PHQ-9 ≥ 14) | 4 (13·8%) | 94 (16·1%) | 98 (16·0%) |
| **Somatic health status** | | | |
| BMI, kg/m^2^ | 24·7 (21·5 to 27·4) | 23·7 (21·5 to 27·4) | 23·7 (21·5 to 27·4) |
| SR Heart disease | 3 (10·0%) | 68 (10·8%) | 71 (10·7%) |
| SR Arterial hypertension | 4 (13·3%) | 117 (18·6%) | 121 (18·4%) |
| SR Hypercholesterolemia | 1 (3·3%) | 38 (6·1%) | 39 (6·0%) |
| SR History of brain infarction | 0 (0·0%) | 30 (4·7%) | 30 (4·5%) |
| SR Diabetes | 2 (6·7%) | 37 (5·9%) | 39 (5·9%) |
| SR Pulmonary disease | 4 (13·3%) | 70 (11·1%) | 74 (11·2%) |
| SR Cancer | 1 (3·4%) | 13 (2·1%) | 14 (2·1%) |
| SR Neurological disease | 2 (6·7%) | 35 (5·6%) | 37 (5·6%) |
| SR Psychological disease | 11 (36·7%) | 138 (22·0%) | 149 (22·7%) |
| SR Liver disease | 8 (26·7%) | 84 (13·4%) | 92 (14·0%) |
| SR HIV | 1 (3·3%) | 6 (1·0%) | 7 (1·1%) |
| SR Tuberculosis | 0 (0·0%) | 8 (1·3%) | 8 (1·2%) |
| **Risk factors** | | | |
| Ever unprotected sex with different partners | 3 (12·5%) | 115 (21·7%) | 118 (21·3%) |
| History of imprisonment | 15 (51·7%) | 324 (54·4%) | 339 (54·2%) |
| Intravenous drug consumption | 8 (26·7%) | 82 (13·6%) | 90 (14·3%) |
| Receiving OAT | 8 (27·6%) | 66 (11·0%) | 74 (11·8%) |
| Alcohol consumption: >7 glasses per week |  |  |  |
| Never | 12 (41·4%) | 201 (33·3%) | 213 (33·6%) |
| Occasional | 9 (31·0%) | 222 (36·8%) | 231 (36·5%) |
| Daily | 8 (27·6%) | 181 (30·0%) | 189 (29·9%) |
| Smoking |  |  |  |
| Never | 5 (17·2%) | 117 (19·3%) | 122 (19·2%) |
| Occasional | 2 (6·9%) | 32 (5·3%) | 34 (5·3%) |
| Daily | 22 (75·9%) | 458 (75·5%) | 480 (75·5%) |
| Ever prescription required medication consumption | 9 (33·3%) | 117 (19·5%) | 126 (20·1%) |
| **Access to healthcare** | | | |
| Having health insurance | 24 (80·0%) | 410 (66·8%) | 434 (67·4%) |
| Visiting a doctor in <12 months | 25 (83·3%) | 440 (72·4%) | 465 (72·9%) |
| Visiting a hospital in <12 months | 11 (36·7%) | 250 (43·2%) | 261 (42·9%) |

**Abbreviations:** PEH, people experiencing homelessness; ETHOS, European typology of homelessness and housing exclusion; EQ5D-VAS, standardised measure for health-related quality of life; UCLA-3, standardised questionnaire for loneliness; GAD-2, standardised questionnaire for anxiety disorders; PHQ-9, standardised questionnaire for depression; BMI, body mass index; SR, self-reported; OAT, opioid agonist treatment.

**Supplementary Table 2.** Clinical characteristics of PEH with active hepatitis B infection, active hepatitis C infection, active hepatitis D infection, and active hepatitis E infection.

|  | **Active HBV**  N=8 | **Active HCV**  N=70 | **Active HDV**  N=1 | **Active HEV**  N=4 |
| --- | --- | --- | --- | --- |
| **Clinical examination** | **Median (IQR) or number (%)** | **Median (IQR) or number (%)** | **Median (IQR) or number (%)** | **Median (IQR) or number (%)** |
| Body mass index, kg/m^2^ | 30·4 (26·3 to 32·4) | 22·1 (20·1 to 25·0) | 23·2 | 34·7 (26·9 to 42·4) |
| Body temperature, °C | 35·5 (35·3 to 36·2) | 36·1 (35·8 to 36·4) | 35·5 | 36·2 (35·7 to 36·6) |
| Fever auricular  Reference: >37.5°C | 0 (0·0) | 0 (0·0) | 0·0 | 0 (0·0) |
| Skin signs of liver damage | 1 (12·5) | 2 (3·2) | 0·0 | 0 (0·0) |
| Weight loss >10% in last 6 months | 0 (0·0) | 9 (14·3) | 0·0 | 1 (25·0) |
| Night sweats | 1 (12·5) | 13 (20·0) | 0·0 | 0 (0·0) |
| Arterial hypertension  Reference: Systolic RR $\geq$140 mmHg and/ or diastolic RR $\geq$90 mmHg | 3 (37·5) | 18 (26·1) | 1·0 | 1 (25·0) |
| **Blood work** |  |  |  |  |
| ***Small blood count*** |  |  |  |  |
| Erythrocytes, mrd/ml  Reference: ♂ 4.8 to 5.9 mrd/ml and  ♀4.3 to 5.2 mg/dl | 5·1 (4·7 to 5.4) | 4·6 (4·3 to 4·9) | 5·2 | 5 (4·5 to 5·1) |
| Thrombocytes, mrd/l  Reference: 150 to 400 mrd/l | 225·0 (153·0 to 266·5) | 245·0 (188·0 to 299·0) | 159·0 | 289·5 (275 to 345·5) |
| Leukocytes, mrd/l  Reference: 4 to 10 mrd/l | 6·7 (5·3 to 9·9) | 7·5 (6·4 to 8·9) | 6·5 | 8·9 (8·0 to 10·0) |
| ***Erythrocytes*** |  |  |  |  |
| MCV, fl  Reference: 85 to 101 fl | 90·8 (89·4 to 93·5) | 91·9 (87·8 to 96·1) | 91·5 | 96·5 (93·3 to 98·8) |
| MCH, pg  Reference: 26 to 34 pg | 30·8 (29·9 to 31·6) | 31·1 (29·7 to 32·1) | 30·8 | 32·3 (31·6 to 33·2) |
| ***For MELD Score*** |  |  |  |  |
| Natrium, mmol/l  Reference: 135-145 mmol/l | 140·5 (139 to 141·5) | 138 (137 to 139) | 142·0 | 139 (136·5 to 141) |
| Bilirubin, mg/dl  Reference: < 1 mg/dl | 0·5 (0·4 to 0·7) | 0·4 (0·3 to 0·6) | 0·5 | 0·5 (0·4 to 0·9) |
| Creatinine, mg/dl  Reference: ♂ 0.5 to 1.1 mg/dl and  ♀0.5 to 0.9 mg/dl | 0·8 (0·8 to 1·0) | 0·8 (0·7 to 0·9) | 0·7 | 0·8 (0·7 to 0·8) |
| MELD score | 6·7 (6·4 to 7·8) | 6·7 (6·4 to 7·4) | ND | 6·7 (6·5 to 7·3) |
| ***Liver enzymes*** |  |  |  |  |
| AST, U/l  Reference: ♂ 10 to 50 mg/dl and  ♀10 to 35 mg/dl | 30 (21 to 40) | 58 (37 to 115) | 37·0 | 17 (15 to 43) |
| ALT, U/l  Reference: ♂ 10 to 45 mg/dl and  ♀10 to 34 mg/dl | 41·5 (24·5 to 64·5) | 58 (36 to 102) | 64·0 | 25 (20 to 29·5) |
| ***Inflammation*** |  |  |  |  |
| CRP, mg/l  Reference: <5 md/l | 4·0 (4·0 to 4·0) | 4·0 (4·0 to 4·4) | 4·0 | 4·0 (4·0 to 4·0) |
| Ferritin, µg/l  Reference: ♂ 34 to 310 mg/dl and  ♀22 to 112 mg/dl | 88·8 (51·1 to 148) | 90·7 (33·1 to 150·7) | 100·7 | 76·5 (54·6 to 135·4) |

**Abbreviation:** PEH, people experiencing homelessness; MCV, mean corpuscular volume; MCH, mean corpuscular haemoglobin; MELD, Model for end-stage liver disease; AST, aspartate aminotransferase; ALT, alanine aminotransferase; CRP, C-reactive protein.

**Supplementary Table 3.** Clinical characteristics of PEH with active HIV infection.

|  | **Active HIV**  N=4 |
| --- | --- |
| **Clinical examination** | **Median (IQR) or number (%)** |
| Body mass index, kg/m^2^ | 19·6 (17·8 to 20·5) |
| Body temperature, °C | 36·2 (35·6 to 36·5) |
| Fever auricular  Reference: >37.5°C | 0 (0·0) |
| Skin signs of liver damage | 0 (0·0) |
| Weight loss >10% in last 6 months | 0 (0·0) |
| Night sweats | 0 (0·0) |
| Arterial hypertension  Reference: Systolic RR $\geq$140 mmHg and/ or diastolic RR $\geq$90 mmHg | 1 (25·0) |
| **Blood work** |  |
| ***Small blood count*** |  |
| Erythrocytes, mrd/ml  Reference: ♂ 4·8 to 5·9 mrd/ml and  ♀4.3 to 5.2 mg/dl | 4·7 (4·5 to 5·2) |
| Thrombocytes, mrd/l  Reference: 150 to 400 mrd/l | 326 (282 to 394) |
| Leukocytes, mrd/l  Reference: 4 to 10 mrd/l | 8·1 (6·2 to 9·6) |
| ***Erythrocytes*** |  |
| MCV, fl  Reference: 85 to 101 fl | 88·4 (86·2 to 92·9) |
| MCH, pg  Reference: 26 to 34 pg | 29·9 (28·2 to 31·6) |
| ***For MELD Score*** |  |
| Natrium, mmol/l  Reference: 135-145 mmol/l | 139·0 (136·5 to 141·5) |
| Bilirubin, mg/dl  Reference: < 1 mg/dl | 0·5 (0·5 to 0·6) |
| Creatinine, mg/dl  Reference: ♂ 0·5 to 1·1 mg/dl and  ♀0.5 to 0.9 mg/dl | 0·8 (0·7 to 0·9) |
| MELD score | ND |
| ***Liver enzymes*** |  |
| AST, U/l  Reference: ♂ 10 to 50 mg/dl and  ♀10 to 35 mg/dl | 26·0 (17·0 to 42·0) |
| ALT, U/l  Reference: ♂ 10 to 45 mg/dl and  ♀10 to 34 mg/dl | 23·5 (15·0 to 34·0) |
| ***Inflammation*** |  |
| CRP, mg/l  Reference: <5 md/l | 4·0 (4·0 to 4·0) |
| Ferritin, µg/l  Reference: ♂ 34 to 310 mg/dl and  ♀22 to 112 mg/dl | 44·8 (14·6 to 96·4) |

**Abbreviation:** PEH, people experiencing homelessness; MCV, mean corpuscular volume; MCH, mean corpuscular haemoglobin; MELD, Model for end-stage liver disease; AST, aspartate aminotransferase; ALT, alanine aminotransferase; CRP, C-reactive protein.

**Supplementary Table 4.** Baseline characteristics of PEH with available and unavailable hepatitis A and B status are illustrated.

|  | **Available HAV and HBV status**  N=586 | **Unavailable HAV and HBV status**  N=57 | **Total**  N=643 |
| --- | --- | --- | --- |
| Age, years | 43·0 (35·0 to 53·0) | 43·0 (38·0 to 51·0) | 43·0 (35·0 to 53·0) |
| Sex |  |  |  |
| Female | 99 (17·5%) | 6 (11·5%) | 105 (17·0%) |
| Male | 468 (82·5%) | 46 (88·5%) | 514 (83·0%) |
| Educational attainment |  |  |  |
| No degree | 96 (17·5%) | 11 (24·4%) | 107 (18·0%) |
| School education | 253 (46·1%) | 23 (51·1%) | 276 (46·5%) |
| Higher education | 200 (36·4%) | 11 (24·4%) | 211 (35·5%) |
| Marital status |  |  |  |
| Single | 366 (66·8%) | 35 (71·4%) | 401 (67·2%) |
| Married | 62 (11·3%) | 7 (14·3%) | 69 (11·6%) |
| Divorced/widowed | 120 (21·9%) | 7 (14·3%) | 127 (21·3%) |
| **Characteristics of homelessness** | | | |
| City of enrolment |  |  |  |
| Frankfurt | 138 (23·5%) | 13 (22·8%) | 151 (23·5%) |
| Hamburg | 175 (29·9%) | 25 (43·9%) | 200 (31·1%) |
| Leipzig | 94 (16·0%) | 7 (12·3%) | 101 (15·7%) |
| Munich | 179 (30·5%) | 12 (21·1%) | 191 (29·7%) |
| ETHOS classification |  |  |  |
| Roofless | 306 (56·5%) | 31 (70·5%) | 337 (57·5%) |
| Homeless | 236 (43·5%) | 13 (29·5%) | 249 (42·5%) |
| Time of homelessness, months | 18·0 (5·0 to 48·0) | 18.0 (3·0 to 72·0) | 18.0 (5·0 to 48·0) |
| Having income from authorities | 250 (45·5%) | 18 (38·3%) | 268 (44·9%) |
| Having income from work | 62 (11·7%) | 6 (13·3%) | 68 (11·9%) |
| **Migration history** | | | |
| Country of birth |  |  |  |
| Germany | 268 (50·6%) | 17 (34·7%) | 285 (49·2%) |
| Abroad | 262 (49·4%) | 32 (65·3%) | 294 (50·8%) |
| Time in Germany, months | 360·0 (72·0 to 540·0) | 144·0 (14·0 to 456·0) | 360·0 (72·0 to 528·0) |
| **Psychiatric health status** | | | |
| EQ-VAS | 75·0 (50·0 to 90·0) | 77·5 (60·0 to 90·0) | 75·0 (50·0 to 90·0) |
| Loneliness according to UCLA-3 | 232 (41·8%) | 21 (43·8%) | 253 (42·0%) |
| Anxiety according to GAD-2 | 153 (27·6%) | 9 (19·1%) | 162 (27·0%) |
| Moderate and severe depression according to PHQ-9 | 88 (16·4%) | 6 (13·3%) | 94 (16·1%) |
| **Somatic health status** | | | |
| BMI, kg/m^2^ | 23·6 (21·5 to 27·1) | 25·2 (22·2 to 29·0) | 23·7 (21·5 to 27·4) |
| SR Heart disease | 61 (10·6%) | 7 (13·0%) | 68 (10·8%) |
| SR Arterial hypertension | 109 (19·0%) | 8 (15·1%) | 117 (18·6%) |
| SR Hypercholesterolemia | 36 (6·3%) | 2 (3·8%) | 38 (6·1%) |
| SR History of brain infarction | 29 (5·0%) | 1 (1·9%) | 30 (4·7%) |
| SR Diabetes | 34 (5·9%) | 3 (5·8%) | 37 (5·9%) |
| SR Pulmonary disease | 65 (11·3%) | 5 (9·6%) | 70 (11·1%) |
| SR Cancer | 13 (2·3%) | 0 (0·0%) | 13 (2·1%) |
| SR Neurological disease | 31 (5·4%) | 4 (7·7%) | 35 (5·6%) |
| SR Psychological disease | 130 (22·7%) | 8 (15·1%) | 138 (22·0%) |
| SR Liver disease | 82 (14·2%) | 2 (3·8%) | 84 (13·4%) |
| SR HIV | 6 (1·0%) | 0 (0·0%) | 6 (1·0%) |
| SR Tuberculosis | 8 (1·4%) | 0 (0·0%) | 8 (1·3%) |
| **Risk factors** | | | |
| Ever unprotected sex with different partners | 101 (20·7%) | 14 (32·6%) | 115 (21·7%) |
| History of imprisonment | 304 (55·3%) | 20 (43·5%) | 324 (54·4%) |
| i.v. drug consumption | 78 (14·1%) | 4 (8·3%) | 82 (13·6%) |
| Time of i.v. drug consumption, months | 120·0 (24·0 to 300·0) | 24·0 (2·0 to 60·0) | 102·0 (22·0 to 294·0) |
| Receiving OAT, months | 64 (11·7%) | 2 (4·0%) | 66 (11·0%) |
| Time of OAT, months | 24·0 (7·0 to 72·0) | 10·0 (10·0 to 10·0) | 24·0 (7·0 to 72·0) |
| Alcohol consumption: >7 glasses per week |  |  |  |
| Never | 178 (32·1%) | 23 (46·0%) | 201 (33·3%) |
| Occasional | 207 (37·4%) | 15 (30·0%) | 222 (36·8%) |
| Daily | 169 (30·5%) | 12 (24·0%) | 181 (30·0%) |
| Time of alcohol consumption, years | 15·0 (3·0 to 25·0) | 12v5 (8·0 to 17·0) | 15·0 (4·0 to 25·0) |
| Smoking |  |  |  |
| Never | 109 (19·5%) | 8 (16·3%) | 117 (19·3%) |
| Occasional | 27 (4·8%) | 5 (10·2%) | 32 (5·3%) |
| Daily | 422 (75·6%) | 36 (73·5%) | 458 (75·5%) |
| Ever prescription required medication consumption | 108 (19·6%) | 9 (18·8%) | 117 (19·5%) |
| **Access to healthcare** | | | |
| Having health insurance status | 385 (68·1%) | 25 (51·0%) | 410 (66·8%) |
| Doctor visit <12 months | 408 (73·1%) | 32 (64·0%) | 440 (72·4%) |
| Hospital visit <12 months | 237 (44·4%) | 13 (28·9%) | 250 (43·2%) |

**Abbreviations:** PEH, people experiencing homelessness; ETHOS, European typology of homelessness and housing exclusion; EQ5D-VAS, standardised measure for health-related quality of life; UCLA-3, standardised questionnaire for loneliness; GAD-2, standardised questionnaire for anxiety disorders; PHQ-9, standardised questionnaire for depression; BMI, body mass index; SR, self-reported; OAT, opioid agonist treatment.

**Supplementary Table 5.** Baseline characteristics in PEH with available and unavailable HCV status are illustrated.

|  | **Available HCV status**  N=584 | **Unavailable HCV status**  N=59 | **Total**  N=643 |
| --- | --- | --- | --- |
| Age, years | 43·0 (35·0 to 53·0) | 43·0 (37·5 to 51·0) | 43·0 (35·0 to 53·0) |
| Sex |  |  |  |
| Female | 98 (17·3%) | 7 (13·0%) | 105 (17·0%) |
| Male | 467 (82·7%) | 47 (87·0%) | 514 (83·0%) |
| Educational attainment |  |  |  |
| No degree | 96 (17·6%) | 11 (23·4%) | 107 (18·0%) |
| School education | 252 (46·1%) | 24 (51·1%) | 276 (46·5%) |
| Higher education | 199 (36·4%) | 12 (25·5%) | 211 (35·5%) |
| Marital status |  |  |  |
| Single | 366 (67·0%) | 35 (68·6%) | 401 (67·2%) |
| Married | 61 (11·2%) | 8 (15·7%) | 69 (11·6%) |
| Divorced/widowed | 119 (21·8%) | 8 (15·7%) | 127 (21·3%) |
| **Characteristics of homelessness** | | | |
| City of enrolment |  |  |  |
| Frankfurt | 137 (23·5%) | 14 (23·7%) | 151 (23·5%) |
| Hamburg | 174 (29·8%) | 26 (44·1%) | 200 (31·1%) |
| Leipzig | 94 (16·1%) | 7 (11·9%) | 101 (15·7%) |
| Munich | 179 (30·7%) | 12 (20·3%) | 191 (29·7%) |
| ETHOS classification |  |  |  |
| Roofless | 306 (56·6%) | 31 (68·9%) | 337 (57·5%) |
| Homeless | 235 (43·4%) | 14 (31·1%) | 249 (42·5%) |
| Time of homelessness, months | 18·0 (5·0 to 48·0) | 24·0 (6·0 to 72·0) | 18·0 (5·0 to 48·0) |
| Having income from authorities | 248 (45·3%) | 20 (40·8%) | 268 (44·9%) |
| Having income from work | 62 (11·8%) | 6 (13·0%) | 68 (11·9%) |
| **Migration history** | | | |
| Country of birth |  |  |  |
| Germany | 268 (50·7%) | 17 (34·0%) | 285 (49·2%) |
| Abroad | 261 (49·3%) | 33 (66·0%) | 294 (50·8%) |
| Time in Germany, months | 366·0 (72·0 to 540·0) | 147·0 (14·0 to 456·0) | 360·0 (72·0 to 528·0) |
| **Psychiatric health status** | | | |
| EQ-VAS | 75·0 (50·0 to 90·0) | 77·5 (60·0 to 90·0) | 75·0 (50·0 to 90·0) |
| Loneliness according to UCLA | 232 (42·0%) | 21 (42·0%) | 253 (42·0%) |
| Anxiety according to GAD-2 | 152 (27·5%) | 10 (20·4%) | 162 (27·0%) |
| Moderate and severe depression according to PHQ-9 | 87 (16·2%) | 7 (14·9%) | 94 (16·1%) |
| **Somatic health status** | | | |
| BMI, kg/m^2^ | 23·6 (21·5 to 27·1) | 25·3 (22·2 to 29·1) | 23·7 (21·5 to 27·4) |
| SR Heart disease | 61 (10·6%) | 7 (12·5%) | 68 (10·8%) |
| SR Arterial hypertension | 109 (19·0%) | 8 (14·5%) | 117 (18·6%) |
| SR Hypercholesterolemia | 36 (6·3%) | 2 (3·7%) | 38 (6·1%) |
| SR History of brain infarction | 29 (5·0%) | 1 (1·8%) | 30 (4·7%) |
| SR Diabetes | 34 (5·9%) | 3 (5·6%) | 37 (5·9%) |
| SR Pulmonary disease | 64 (11·1%) | 6 (11·1%) | 70 (11·1%) |
| SR Cancer | 13 (2·3%) | 0 (0·0%) | 13 (2·1%) |
| SR Neurological disease | 30 (5·2%) | 5 (9·3%) | 35 (5·6%) |
| SR Psychological disease | 129 (22·6%) | 9 (16·4%) | 138 (22·0%) |
| SR Liver disease | 82 (14·3%) | 2 (3·6%) | 84 (13·4%) |
| SR HIV | 5 (0·9%) | 1 (1·9%) | 6 (1·0%) |
| SR Tuberculosis | 8 (1·4%) | 0 (0·0%) | 8 (1·3%) |
| **Risk factors** | | | |
| Ever unprotected sex with different partners | 101 (20·8%) | 14 (31·1%) | 115 (21·7%) |
| History of imprisonment | 302 (55·1%) | 22 (45·8%) | 324 (54·4%) |
| i.v. drug consumption | 77 (14·0%) | 5 (10·0%) | 82 (13·6%) |
| Time of i.v. drug consumption, months | 120·0 (22·0 to 300·0) | 24·0 (13·0 to 42·0) | 102·0 (22·0 to 294·0) |
| Receiving OAT | 63 (11·5%) | 3 (5·8%) | 66 (11·0%) |
| Time receiving OAT, month | 24·0 (7·0 to 60·0) | 65·0 (10·0 to 120·0) | 24·0 (7·0 to 72·0) |
| Alcohol consumption: >7 glasses per week |  |  |  |
| Never | 177 (32·1%) | 24 (46·2%) | 201 (33·3%) |
| Occasional | 206 (37·3%) | 16 (30·8%) | 222 (36·8%) |
| Daily | 169 (30·6%) | 12 (23·1%) | 181 (30·0%) |
| Time of alcohol consumption, years | 15·0 (3·0 to 25·0) | 12·5 (8·0 to 17·0) | 15·0 (4·0 to 25·0) |
| Smoking |  |  |  |
| Never | 109 (19·6%) | 8 (15·7%) | 117 (19·3%) |
| Occasional | 27 (4·9%) | 5 (9·8%) | 32 (5·3%) |
| Daily | 420 (75·5%) | 38 (74·5%) | 458 (75·5%) |
| Ever prescription required medication consumption | 107 (19·5%) | 10 (20·0%) | 117 (19·5%) |
| **Access to healthcare** | | | |
| Having health insurance status | 384 (68·2%) | 26 (51·0%) | 410 (66·8%) |
| Doctor visit <12 months | 407 (73·2%) | 33 (63·5%) | 440 (72·4%) |
| Hospital visit <12 months | 235 (44·2%) | 15 (31·9%) | 250 (43·2%) |

**Abbreviations:** PEH, people experiencing homelessness; LCA, latent class analysis; ETHOS, European typology of homelessness and housing exclusion; EQ5D-VAS, standardised measure for health-related quality of life; UCLA-3, standardised questionnaire for loneliness; GAD-2, standardised questionnaire for anxiety disorders; PHQ-9, standardised questionnaire for depression; BMI, body mass index; SR, self-reported; OAT, opioid antagonist treatment.

**Supplementary Table 6.** Baseline characteristics in PEH with available and unavailable hepatitis E status are illustrated.

|  | **Available HEV status**  N=584 | **Unavailable HEV status**  N=59 | **Total**  N=643 |
| --- | --- | --- | --- |
| Age, years | 43·0 (35·0 to 53·0) | 43·0 (37·5 to 51·5) | 43·0 (35·0 to 53·0) |
| Sex |  |  |  |
| Female | 97 (17·2%) | 8 (14·8%) | 105 (17·0%) |
| Male | 468 (82·8%) | 46 (85·2%) | 514 (83·0%) |
| Educational attainment |  |  |  |
| No degree | 96 (17·6%) | 11 (23·4%) | 107 (18·0%) |
| School education | 252 (46·1%) | 24 (51·1%) | 276 (46·5%) |
| Higher education | 199 (36·4%) | 12 (25·5%) | 211 (35·5%) |
| Marital status |  |  |  |
| Single | 365 (66·7%) | 36 (72·0%) | 401 (67·2%) |
| Married | 62 (11·3%) | 7 (14·0%) | 69 (11·6%) |
| Divorced/widowed | 120 (21·9%) | 7 (14·0%) | 127 (21·3%) |
| **Characteristics of homelessness** | | | |
| City of enrolment |  |  |  |
| Frankfurt | 138 (23·6%) | 13 (22·0%) | 151 (23·5%) |
| Hamburg | 175 (30·0%) | 25 (42·4%) | 200 (31·1%) |
| Leipzig | 92 (15·8%) | 9 (15·3%) | 101 (15·7%) |
| Munich | 179 (30·7%) | 12 (20·3%) | 191 (29·7%) |
| ETHOS classification |  |  |  |
| Roofless | 304 (56·3%) | 33 (71·7%) | 337 (57·5%) |
| Homeless | 236 (43·7%) | 13 (28·3%) | 249 (42·5%) |
| Time of homelessness, months | 18·0 (5·0 to 48·0) | 14·0 (3·0 to 72·0) | 18·0 (5·0 to 48·0) |
| Having income from authorities | 248 (45·3%) | 20 (40·8%) | 268 (44·9%) |
| Having income from work | 62 (11·8%) | 6 (12·8%) | 68 (11·9%) |
| **Migration history** | | | |
| Country of birth |  |  |  |
| Germany | 266 (50·4%) | 19 (37·3%) | 285 (49·2%) |
| Abroad | 262 (49·6%) | 32 (62·7%) | 294 (50·8%) |
| Time in Germany, months | 360·0 (72·0 to 540·0) | 150·0 (14·0 to 480·0) | 360·0 (72·0 to 528·0) |
| **Psychiatric health status** | | | |
| EQ-VAS | 75·0 (50·0 to 90·0) | 75·0 (60·0 to 90·0) | 75·0 (50·0 to 90·0) |
| Loneliness according to UCLA | 232 (42·0%) | 21 (42·0%) | 253 (42·0%) |
| Anxiety according to GAD-2 | 153 (27·7%) | 9 (18·4%) | 162 (27·0%) |
| Moderate and severe depression according to PHQ-9 | 88 (16·4%) | 6 (12·8%) | 94 (16·1%) |
| **Somatic health status** | | | |
| BMI, kg/m^2^ | 23·6 (21·5 to 27·1) | 25·3 (22·2 to 29·1) | 23·7 (21·5 to 27·4) |
| SR Heart disease | 60 (10·4%) | 8 (14·5%) | 68 (10·8%) |
| SR Arterial hypertension | 108 (18·8%) | 9 (16·7%) | 117 (18·6%) |
| SR Hypercholesterolemia | 35 (6·1%) | 3 (5·7%) | 38 (6·1%) |
| SR History of brain infarction | 29 (5·0%) | 1 (1·8%) | 30 (4·7%) |
| SR Diabetes | 33 (5·8%) | 4 (7·5%) | 37 (5·9%) |
| SR Pulmonary disease | 65 (11·3%) | 5 (9·3%) | 70 (11·1%) |
| SR Cancer | 13 (2·3%) | 0 (0·0%) | 13 (2·1%) |
| SR Neurological disease | 31 (5·4%) | 4 (7·4%) | 35 (5·6%) |
| SR Psychological disease | 129 (22·6%) | 9 (16·4%) | 138 (22·0%) |
| SR Liver disease | 82 (14·3%) | 2 (3·6%) | 84 (13·4%) |
| SR HIV | 6 (1·0%) | 0 (0·0%) | 6 (1·0%) |
| SR Tuberculosis | 8 (1·4%) | 0 (0·0%) | 8 (1·3%) |
| **Risk factors** | | | |
| Ever unprotected sex with different partners | 101 (20·8%) | 14 (31·1%) | 115 (21·7%) |
| History of imprisonment | 304 (55·5%) | 20 (41·7%) | 324 (54·4%) |
| i.v. drug consumption | 77 (14·0%) | 5 (10·0%) | 82 (13·6%) |
| Time of i.v. drug consumption, months | 120·0 (24·0 to 300·0) | 15·0 (4·0 to 42·0) | 102·0 (22·0 to 294·0) |
| Receiving OAT | 64 (11·7%) | 2 (3·8%) | 66 (11·0%) |
| Time of OAT, months | 24·0 (7·0 to 72·0) | 10·0 (10·0 to 10·0) | 24·0 (7·0 to 72·0) |
| Alcohol consumption: >7 glasses per week |  |  |  |
| Never | 178 (32·2%) | 23 (44.2%) | 201 (33·3%) |
| Occasional | 205 (37·1%) | 17 (32·7%) | 222 (36·8%) |
| Daily | 169 (30·6%) | 12 (23·1%) | 181 (30·0%) |
| Time of alcohol consumption, years | 15·0 (3·0 to 25·0) | 12·5 (8·0 to 17·0) | 15·0 (4·0 to 25·0) |
| Smoking |  |  |  |
| Never | 108 (19·4%) | 9 (17·6%) | 117 (19·3%) |
| Occasional | 27 (4·9%) | 5 (9·8%) | 32 (5·3%) |
| Daily | 421 (75·7%) | 37 (72·5%) | 458 (75·5%) |
| Ever prescription required medication consumption | 108 (19·6%) | 9 (18·0%) | 117 (19·5%) |
| **Access to healthcare** | | | |
| Having health insurance status | 383 (68·0%) | 27 (52·9%) | 410 (66·8%) |
| Doctor visit <12 months | 406 (73·0%) | 34 (65·4%) | 440 (72·4%) |
| Hospital visit <12 months | 237 (44·5%) | 13 (27·7%) | 250 (43·2%) |

**Abbreviations:** PEH, people experiencing homelessness; ETHOS, European typology of homelessness and housing exclusion; EQ5D-VAS, standardised measure for health-related quality of life; UCLA-3, standardised questionnaire for loneliness; GAD-2, standardised questionnaire for anxiety disorders; PHQ-9, standardised questionnaire for depression; BMI, body mass index; SR, self-reported; OAT, opioid antagonist treatment.

**Supplementary Table 7.** Baseline characteristics in PEH with available and unavailable HIV status are illustrated.

|  | **Available HIV status**  N=605 | **Unavailable HIV status**  N=38 | **Total**  N=643 |
| --- | --- | --- | --- |
| Age, years | 43·0 (35·0 to 53·0) | 42·0 (37·0 to 49·5) | 43·0 (35·0 to 53·0) |
| Sex |  |  |  |
| Female | 100 (17·2%) | 5 (13·9%) | 105 (17·0%) |
| Male | 483 (82·8%) | 31 (86·1%) | 514 (83·0%) |
| Educational attainment |  |  |  |
| No degree | 99 (17·7%) | 8 (23·5%) | 107 (18·0%) |
| School education | 262 (46·8%) | 14 (41·2%) | 276 (46·5%) |
| Higher education | 199 (35·5%) | 12 (35·3%) | 211 (35·5%) |
| Marital status |  |  |  |
| Single | 374 (66·5%) | 27 (77·1%) | 401 (67·2%) |
| Married | 66 (11·7%) | 3 (8·6%) | 69 (11·6%) |
| Divorced/widowed | 122 (21·7%) | 5 (14·3%) | 127 (21·3%) |
| **Characteristics of homelessness** | | | |
| City of enrolment |  |  |  |
| Frankfurt | 146 (24·1%) | 5 (13·2%) | 151 (23·5%) |
| Hamburg | 182 (30·1%) | 18 (47·4%) | 200 (31·1%) |
| Leipzig | 95 (15·7%) | 6 (15·8%) | 101 (15·7%) |
| Munich | 182 (30·1%) | 9 (23·7%) | 191 (29·7%) |
| ETHOS classification |  |  |  |
| Roofless | 317 (57·1%) | 20 (64·5%) | 337 (57·5%) |
| Homeless | 238 (42·9%) | 11 (35·5%) | 249 (42·5%) |
| Time of homelessness, months | 18·0 (5·0 to 48·0) | 14·0 (6·0 to 48·0) | 18·0 (5·0 to 48·0) |
| Having income from authorities | 252 (44·9%) | 16 (44·4%) | 268 (44·9%) |
| Having income from work | 62 (11·5%) | 6 (17·1%) | 68 (11·9%) |
| **Migration history** | | | |
| Country of birth |  |  |  |
| Germany | 266 (49·0%) | 19 (52·8%) | 285 (49·2%) |
| Abroad | 277 (51·0%) | 17 (47·2%) | 294 (50·8%) |
| Time in Germany, months | 360·0 (72·0 to 528·0) | 342·0 (22·0 to 504·0) | 360·0 (72·0 to 528·0) |
| **Psychiatric health status** | | | |
| EQ-VAS | 75·0 (50·0 to 90·0) | 80·0 (55·5 to 90·0) | 75·0 (50·0 to 90·0) |
| Loneliness according to UCLA-3 | 239 (42·2%) | 14 (38·9%) | 253 (42·0%) |
| Anxiety according to GAD-2 | 156 (27·5%) | 6 (17·6%) | 162 (27·0%) |
| Moderate and severe depression according to PHQ-9 | 92 (16·8%) | 2 (5·9%) | 94 (16·1%) |
| **Somatic health status** | | | |
| BMI, kg/m^2^ | 23·7 (21·5 to 27·1) | 25·2 (21·9 to 28·9) | 23·7 (21·5 to 27·4) |
| SR Heart disease | 64 (10·8%) | 4 (10·5%) | 68 (10·8%) |
| SR Arterial hypertension | 111 (18·8%) | 6 (15·8%) | 117 (18·6%) |
| SR Hypercholesterolemia | 36 (6·1%) | 2 (5·3%) | 38 (6·1%) |
| SR History of brain infarction | 30 (5·0%) | 0 (0·0%) | 30 (4·7%) |
| SR Diabetes | 34 (5·8%) | 3 (7·9%) | 37 (5·9%) |
| SR Pulmonary disease | 67 (11·3%) | 3 (7·9%) | 70 (11·1%) |
| SR Cancer | 13 (2·2%) | 0 (0·0%) | 13 (2·1%) |
| SR Neurological disease | 32 (5·4%) | 3 (7·9%) | 35 (5·6%) |
| SR Psychological disease | 131 (22·3%) | 7 (18·4%) | 138 (22·0%) |
| SR Liver disease | 82 (13·9%) | 2 (5·3%) | 84 (13·4%) |
| SR HIV | 6 (1·0%) | 0 (0·0%) | 6 (1·0%) |
| SR Tuberculosis | 8 (1·4%) | 0 (0·0%) | 8 (1·3%) |
| **Risk factors** | | | |
| Ever unprotected sex with different partners | 104 (20·9%) | 11 (33·3%) | 115 (21·7%) |
| History of imprisonment | 311 (55·2%) | 13 (39·4%) | 324 (54·4%) |
| i.v. drug consumption | 81 (14·3%) | 1 (2·9%) | 82 (13·6%) |
| Time of i.v. drug consumption, months | 120·0 (24·0 to 300·0) | 2·0 (2·0 to 2·0) | 102·0 (22·0 to 294·0) |
| Receiving OAT | 64 (11·4%) | 2 (5·6%) | 66 (11·0%) |
| Time of OAT, months | 21·0 (7·0 to 72·0) | 24·0 (24·0 to 24·0) | 24·0 (7·0 to 72·0) |
| Alcohol consumption: >7 glasses per week |  |  |  |
| Never | 186 (32·8%) | 15 (40·5%) | 201 (33.3%) |
| Occasional | 208 (36·7%) | 14 (37·8%) | 222 (36.8%) |
| Daily | 173 (30·5%) | 8 (21·6%) | 181 (30.0%) |
| Time of alcohol consumption, years | 15·0 (3·0 to 25·0) | 15·0 (8·0 to 20·0) | 15·0 (40 to 25·0) |
| Smoking |  |  |  |
| Never | 110 (19·3%) | 7 (19·4%) | 117 (19·3%) |
| Occasional | 29 (5·1%) | 3 (8·3%) | 32 (5·3%) |
| Daily | 432 (75·7%) | 26 (72·2%) | 458 (75·5%) |
| Ever prescription required medication consumption | 111 (19·6%) | 6 (17·1%) | 117 (19·5%) |
| **Access to healthcare** | | | |
| Having health insurance status | 387 (67·0%) | 23 (63·9%) | 410 (66·8%) |
| Doctor visit <12 months | 416 (72·7%) | 24 (66·7%) | 440 (72·4%) |
| Hospital visit <12 months | 241 (44·4%) | 9 (25·0%) | 250 (43·2%) |

**Abbreviations:** PEH, people experiencing homelessness; ETHOS, European typology of homelessness and housing exclusion; EQ5D-VAS, standardised measure for health-related quality of life; UCLA-3, standardised questionnaire for loneliness; GAD-2, standardised questionnaire for anxiety disorders; PHQ-9, standardised questionnaire for depression; BMI, body mass index; SR, self-reported; OAT, opioid agonist treatment.

**
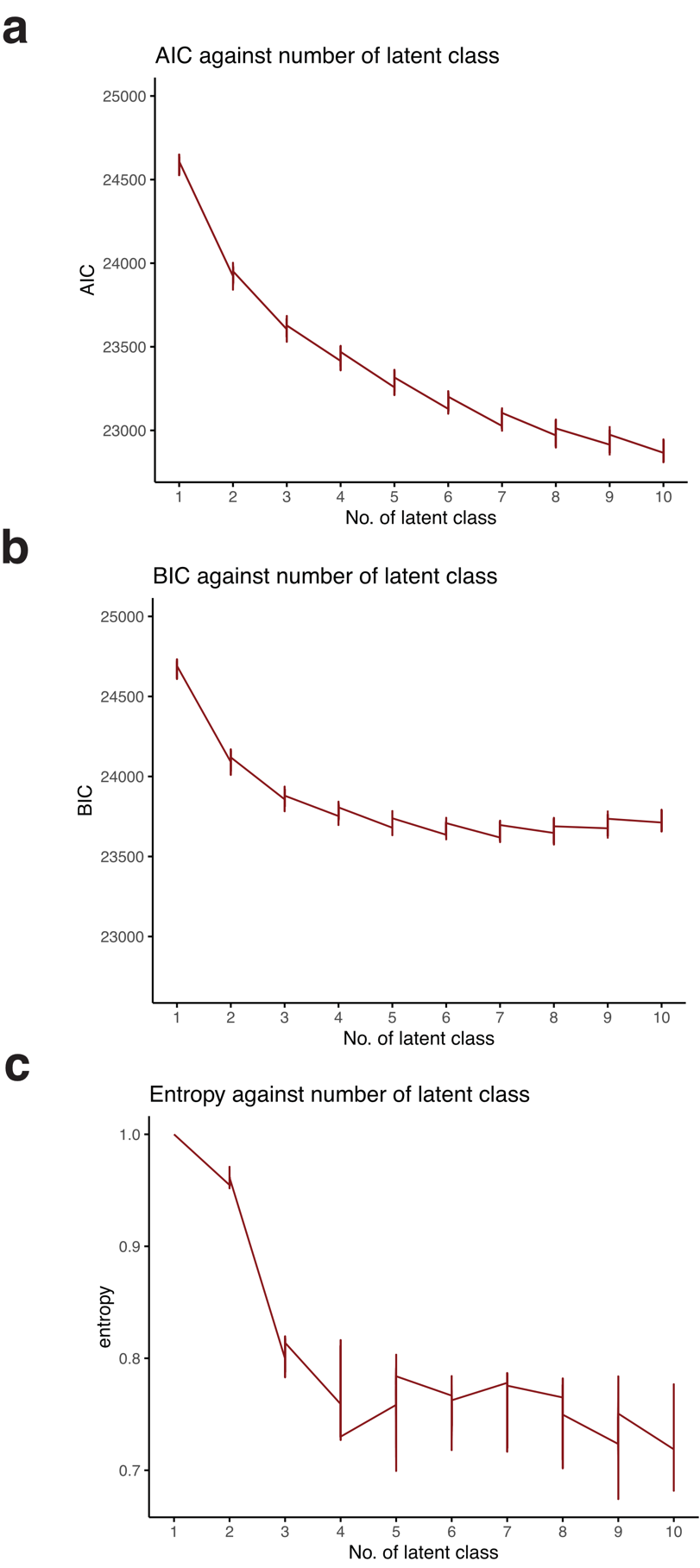
Supplementary Figure 1.** Latent class fit statistics by the number of latent classes.

**Abbreviations:** AIC, Akaike’s information criterion; BIC, Bayesian information criterion.


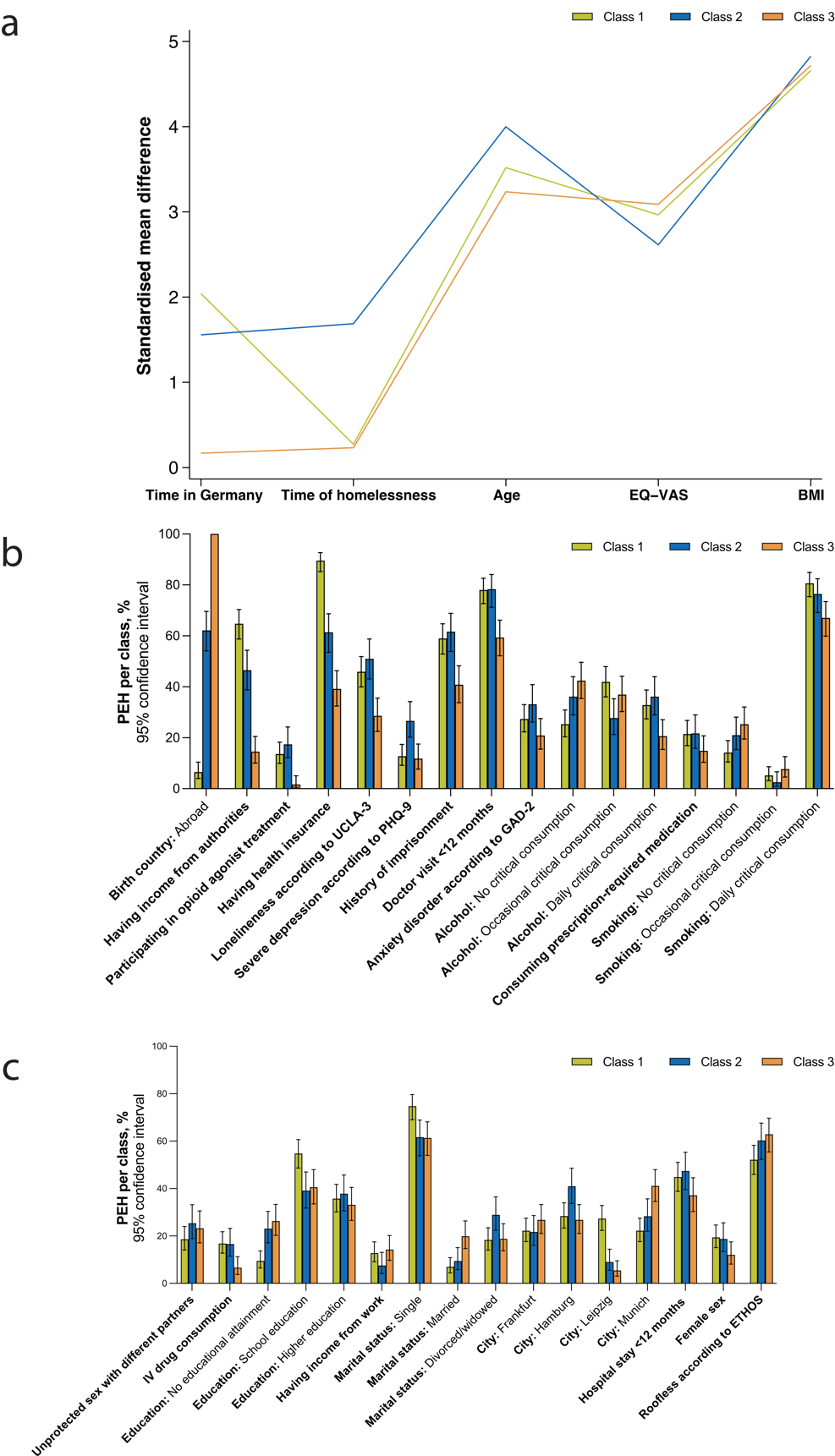
**Supplementary Figure 2.** The standardised mean of continuous variables by class (**a**) and frequencies of categorical variables by class (**b** and **c**) are illustrated with decreasing degree of separation between classes. Variables were sorted by the mean squares of error from ANOVA.

**Abbreviations:** PEH, people experiencing homelessness; ETHOS, European typology of homelessness and housing exclusion; EQ5D-VAS, standardised measure for health-related quality of life; UCLA-3, standardised questionnaire for loneliness; GAD-2, standardised questionnaire for anxiety disorders; PHQ-9, standardised questionnaire for depression; BMI, body mass index; SR, self-reported;; ANOVA, analysis of variance.

**Supplementary Figure 3.** Venn diagram for overlap between active HIV and active or past hepatitis infections. Non-missing percentages are shown for cases with complete measurements for all infections illustrated (n=579).


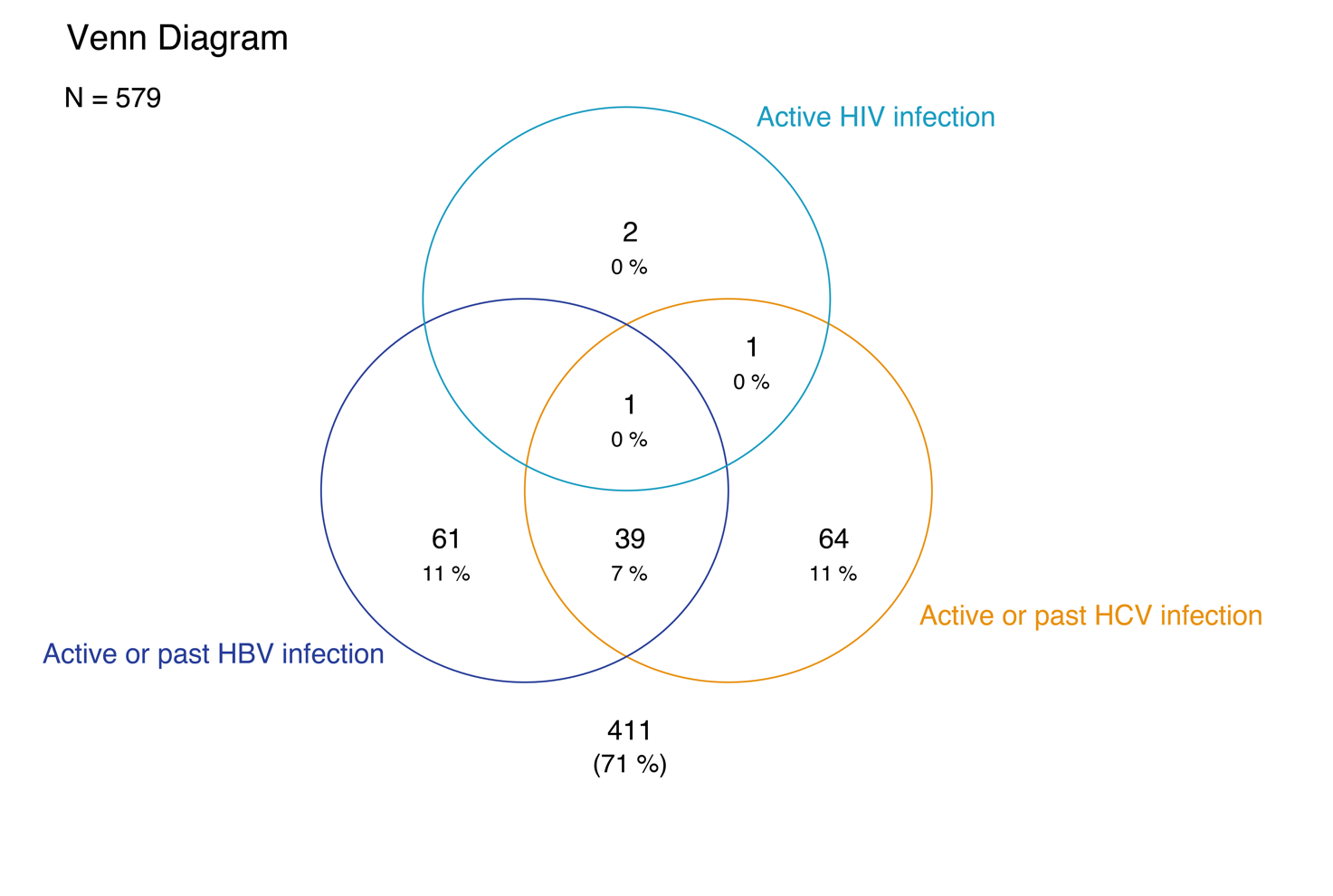


**Supplementary Figure 4.** Numbers of PEH in the German homeless population according to the country of birth.

**
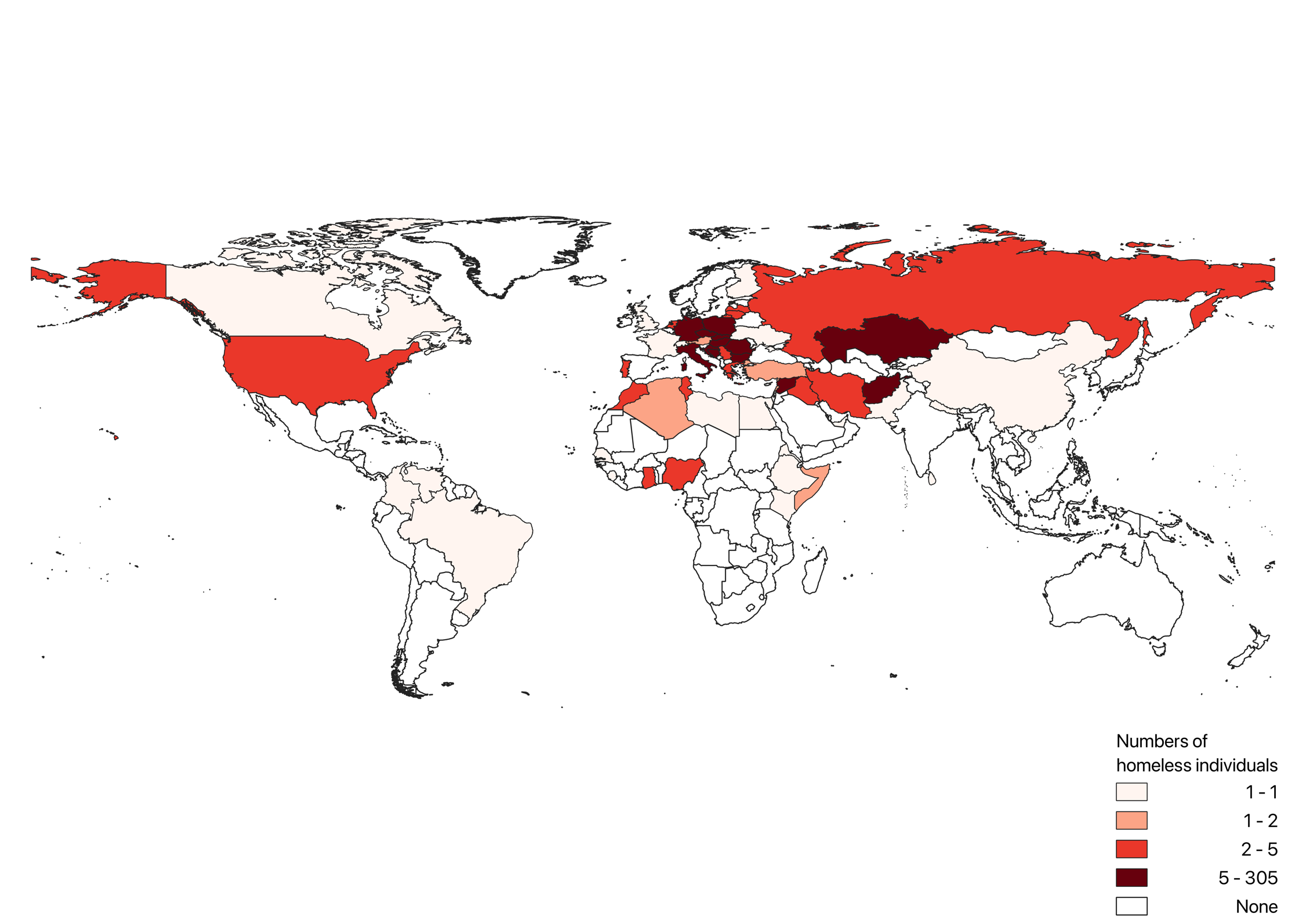
**


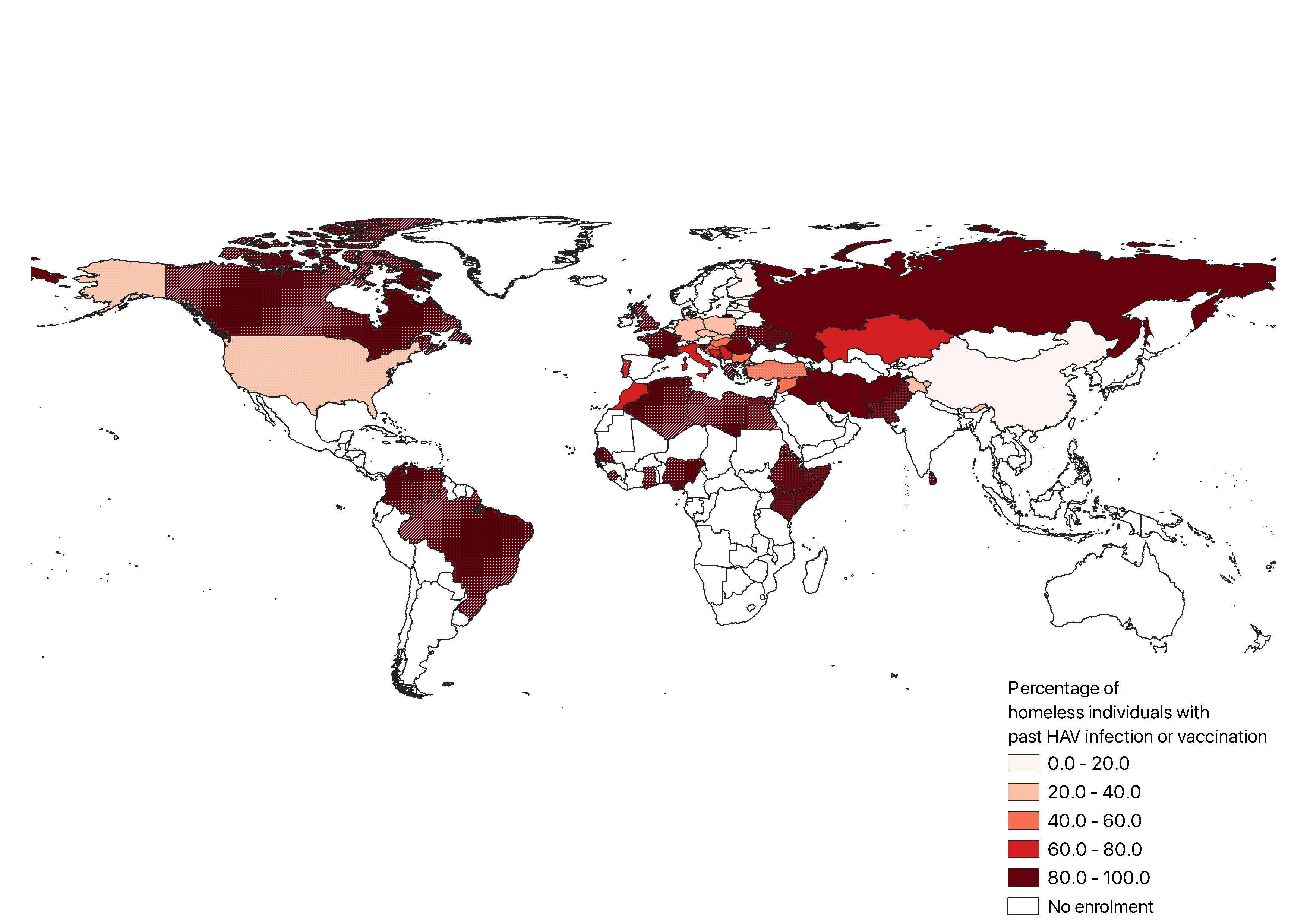
**Supplementary Figure 5.** Percentage of PEH diagnosed with past HAV infection and/or vaccination according to the country of birth. White hatching illustrates enrolment numbers below 5.


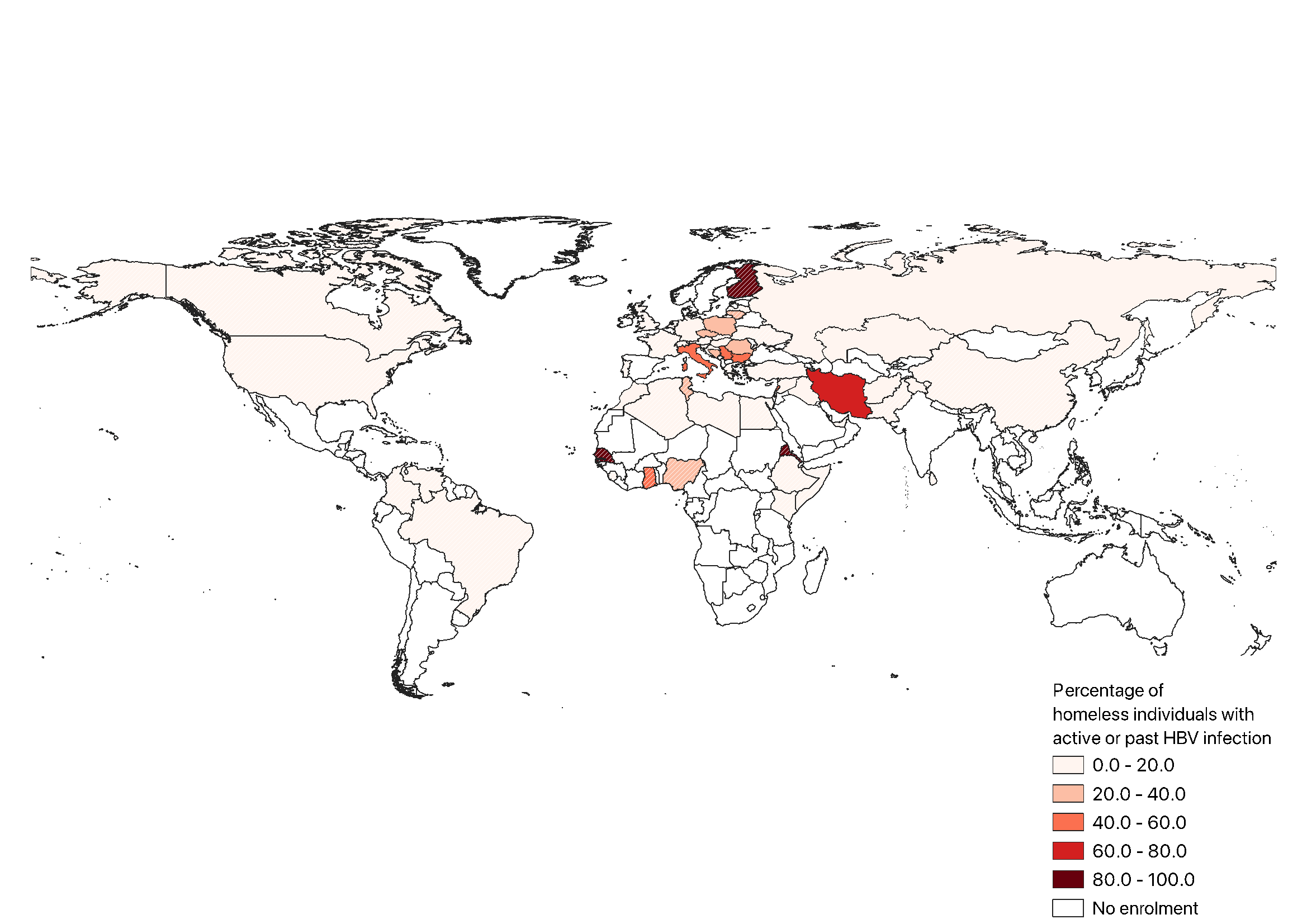
**Supplementary Figure 6.** Percentage of PEH diagnosed with active or past HBV infection in the German homeless population according to the country of birth. White hatching illustrates enrolment numbers below 5.


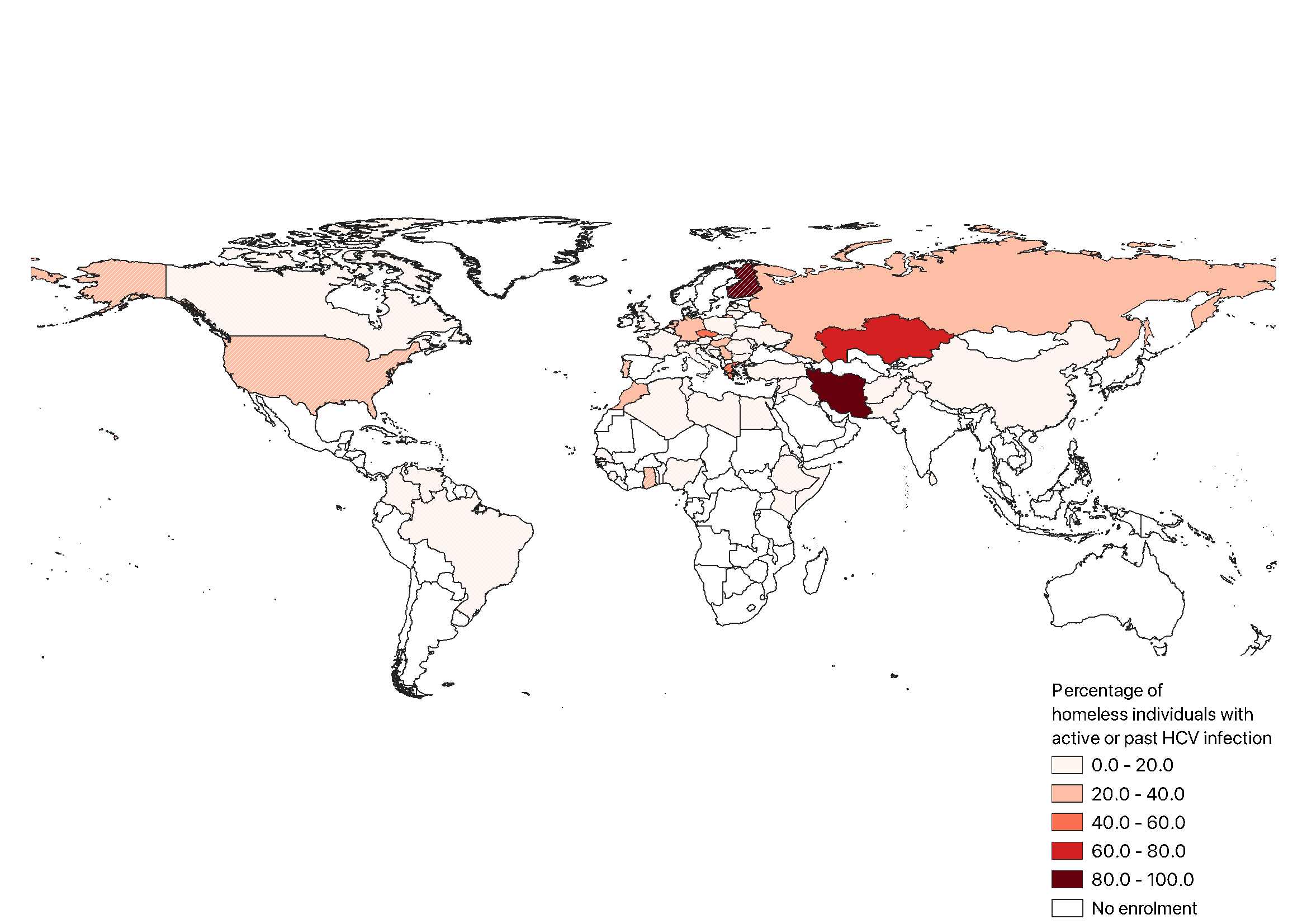
**Supplementary Figure 7.** Percentage of PEH diagnosed with active or past HCV infection in the German homeless population according to the country of birth. White hatching illustrates enrolment numbers below 5.

**
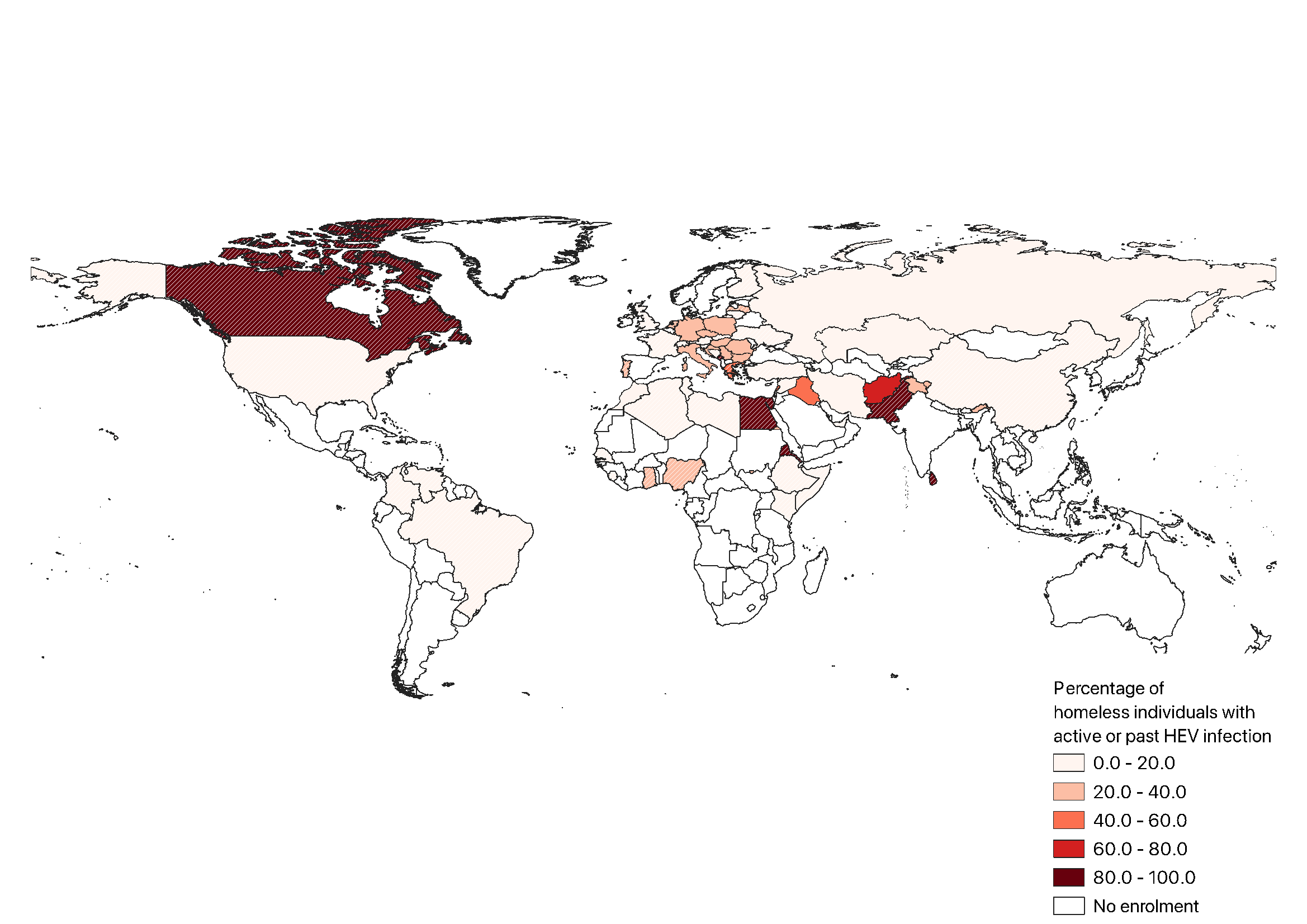
Supplementary Figure 8.** Percentage of PEH diagnosed with active or past HEV infection in the German homeless population according to the country of birth. White hatching illustrates enrolment numbers below 5.

**Supplementary Figure 9.** Percentage of PEH diagnosed with HBV vaccination among individuals without active or past HBV infection in the German homeless population according to the country of birth. White hatching illustrates enrolment numbers below 5.


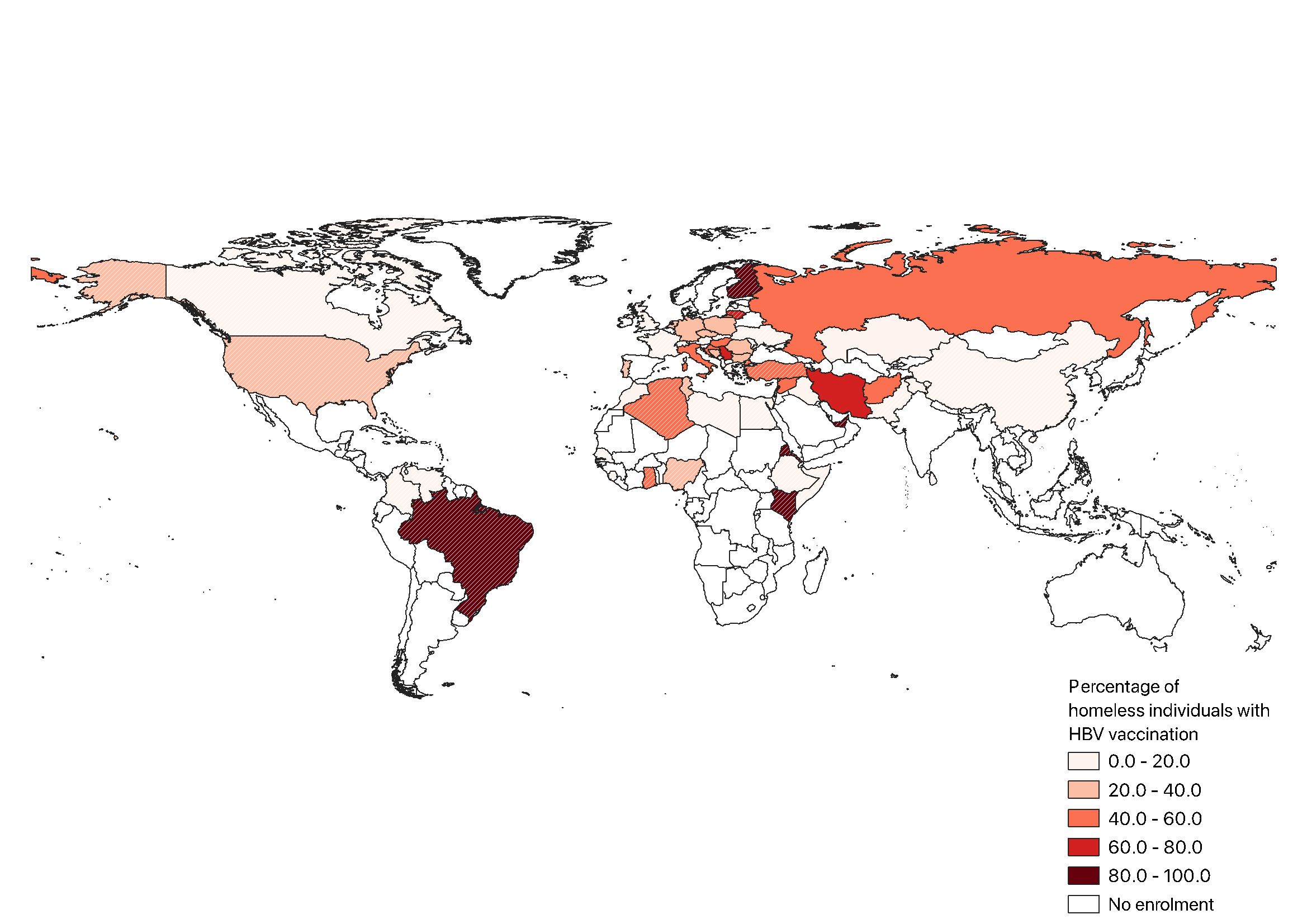


**
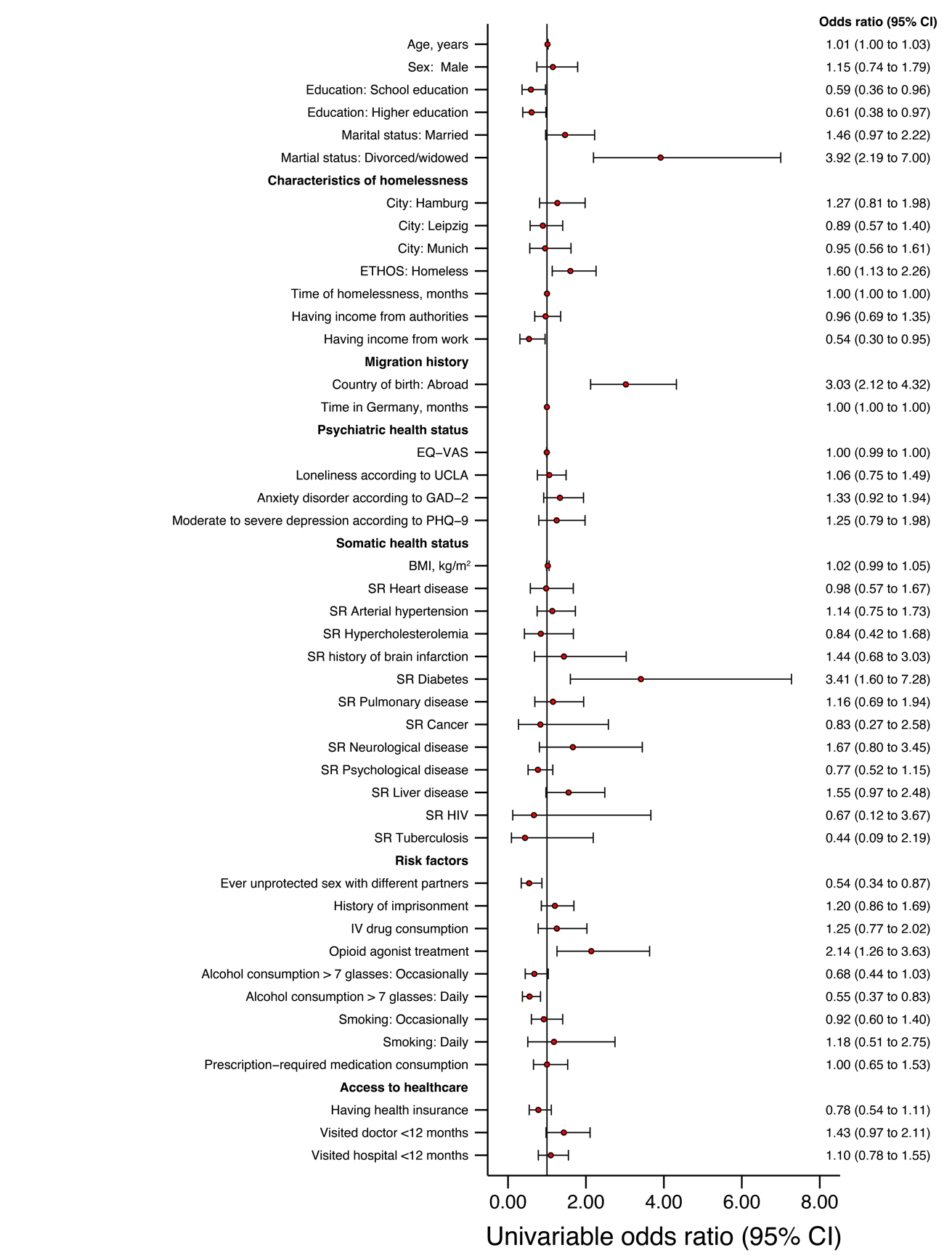
Supplementary Figure 10.** Odds ratios and 95% confidence intervals with past HAV infection or vaccination as the model-dependent variable are illustrated.

**Abbreviations:** ETHOS, European typology of homelessness and housing exclusion; EQ5D-VAS, standardised measure for health-related quality of life; UCLA-3, standardised questionnaire for loneliness; GAD-2, standardised questionnaire for anxiety disorders; PHQ-9, standardised questionnaire for depression; BMI, body mass index; SR, self-reported; OAT, opioid agonist therapy. **Supplementary Figure 11.** Odds ratios and 95% confidence intervals with active or past HBV **
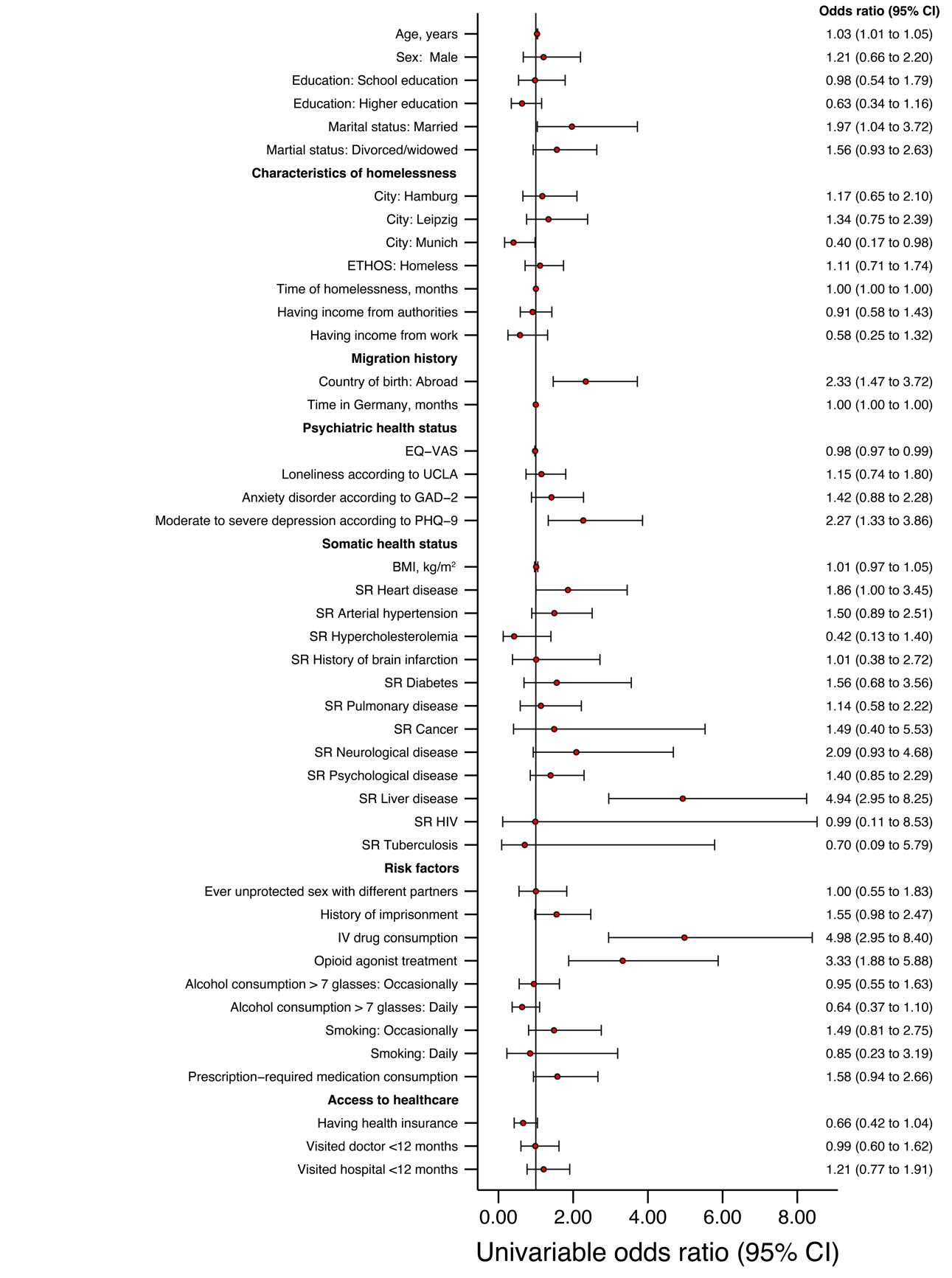
**infection as the model-dependent variable are illustrated.

**Abbreviations:** ETHOS, European typology of homelessness and housing exclusion; EQ5D-VAS, standardised measure for health-related quality of life; UCLA-3, standardised questionnaire for loneliness; GAD-2, standardised questionnaire for anxiety disorders; PHQ-9, standardised questionnaire for depression; BMI, body mass index; SR, self-reported; OAT, opioid agonist therapy.


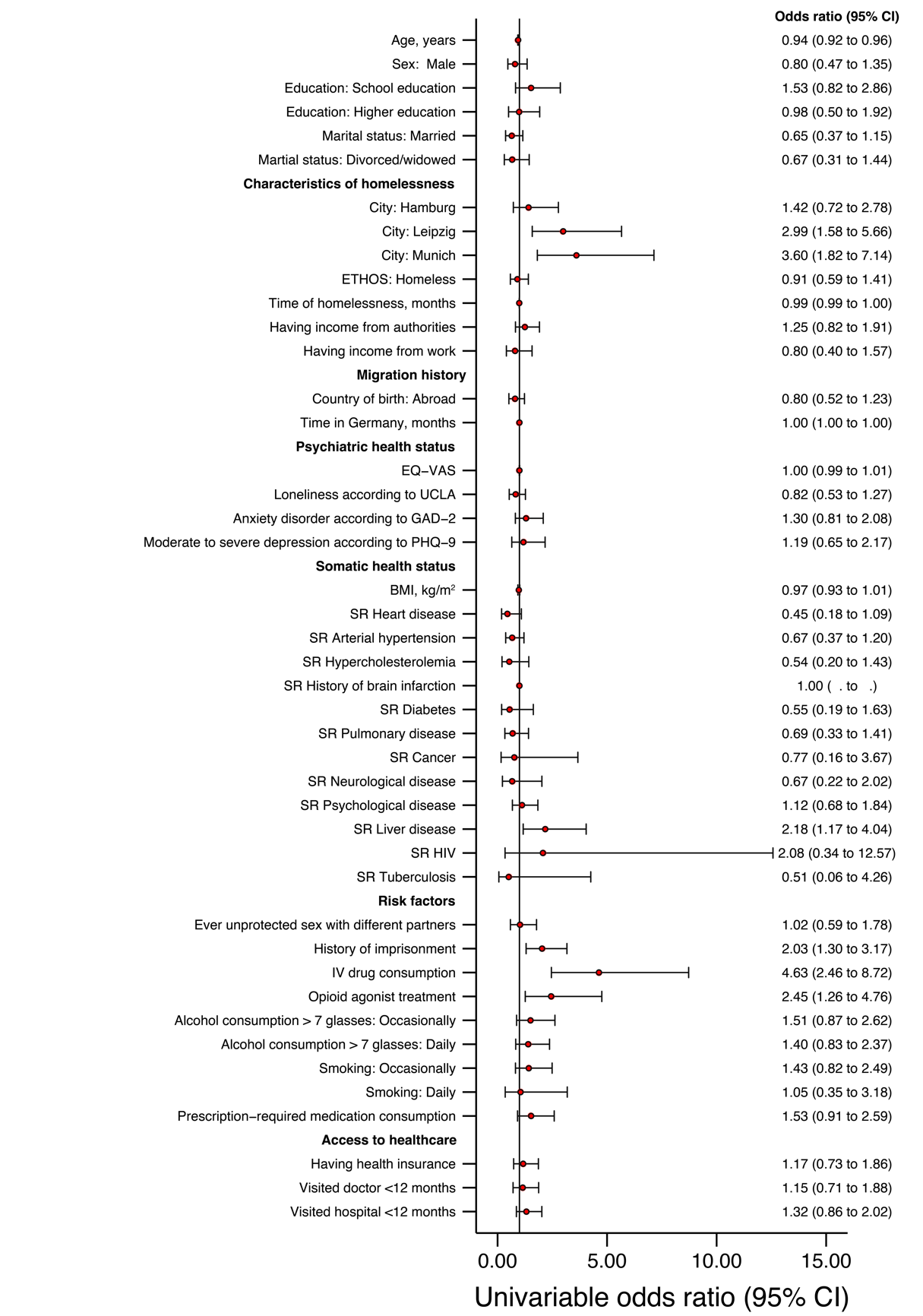
**Supplementary Figure 12.** Odds ratios and 95% confidence intervals with HBV vaccination in homeless individuals without active or past HBV infection as the model-dependent variable are illustrated.

**Abbreviations:** ETHOS, European typology of homelessness and housing exclusion; EQ5D-VAS, standardised measure for health-related quality of life; UCLA-3, standardised questionnaire for loneliness; GAD-2, standardised questionnaire for anxiety disorders; PHQ-9, standardised questionnaire for depression; BMI, body mass index; SR, self-reported; OAT, opioid agonist therapy.


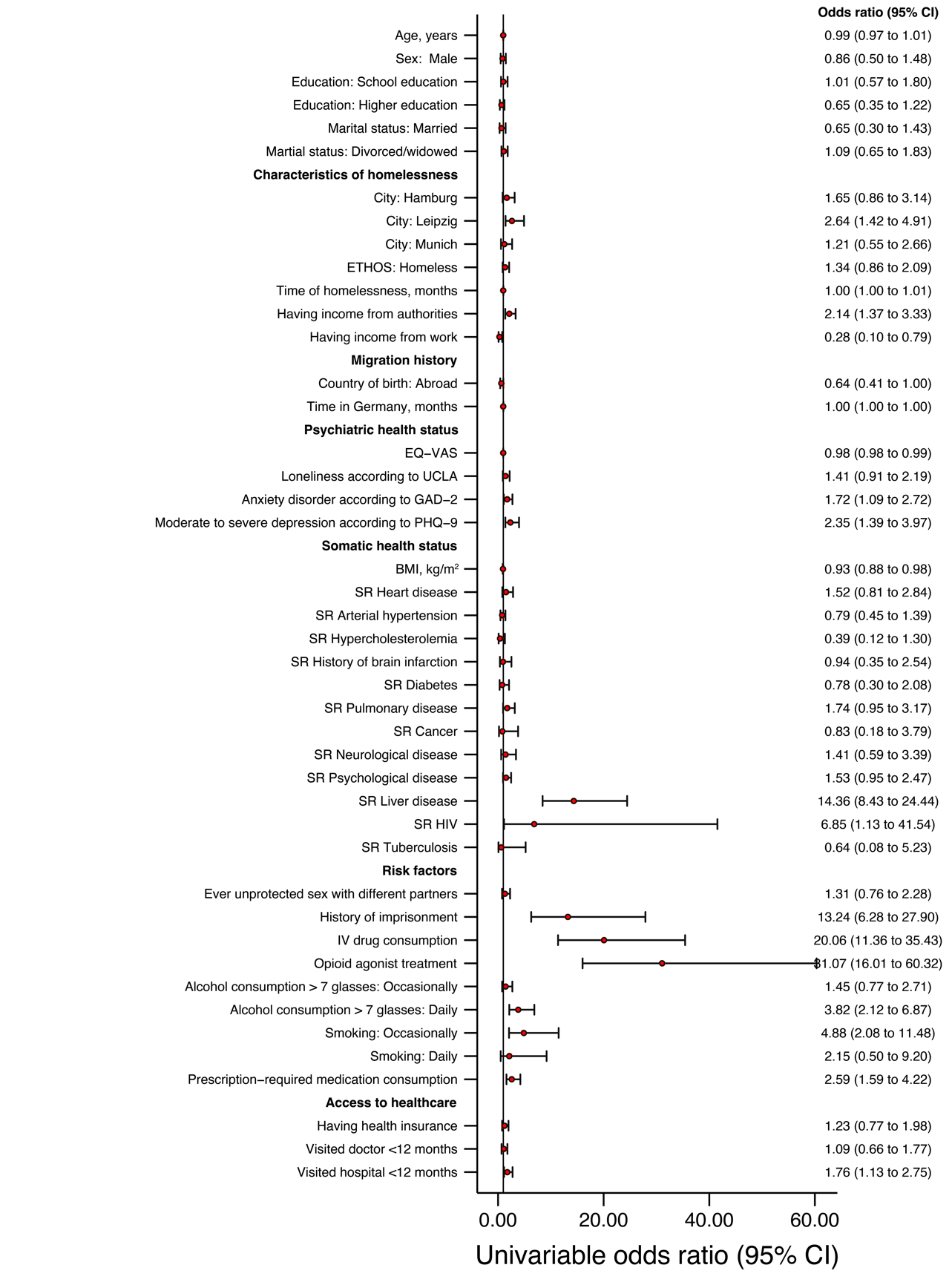
**Supplementary Figure 13.** Odds ratios and 95% confidence intervals with active or past HCV infection as the model-dependent variable are illustrated.

**Abbreviations:** ETHOS, European typology of homelessness and housing exclusion; EQ5D-VAS, standardised measure for health-related quality of life; UCLA-3, standardised questionnaire for loneliness; GAD-2, standardised questionnaire for anxiety disorders; PHQ-9, standardised questionnaire for depression; BMI, body mass index; SR, self-reported, OAT, opioid agonist therapy.


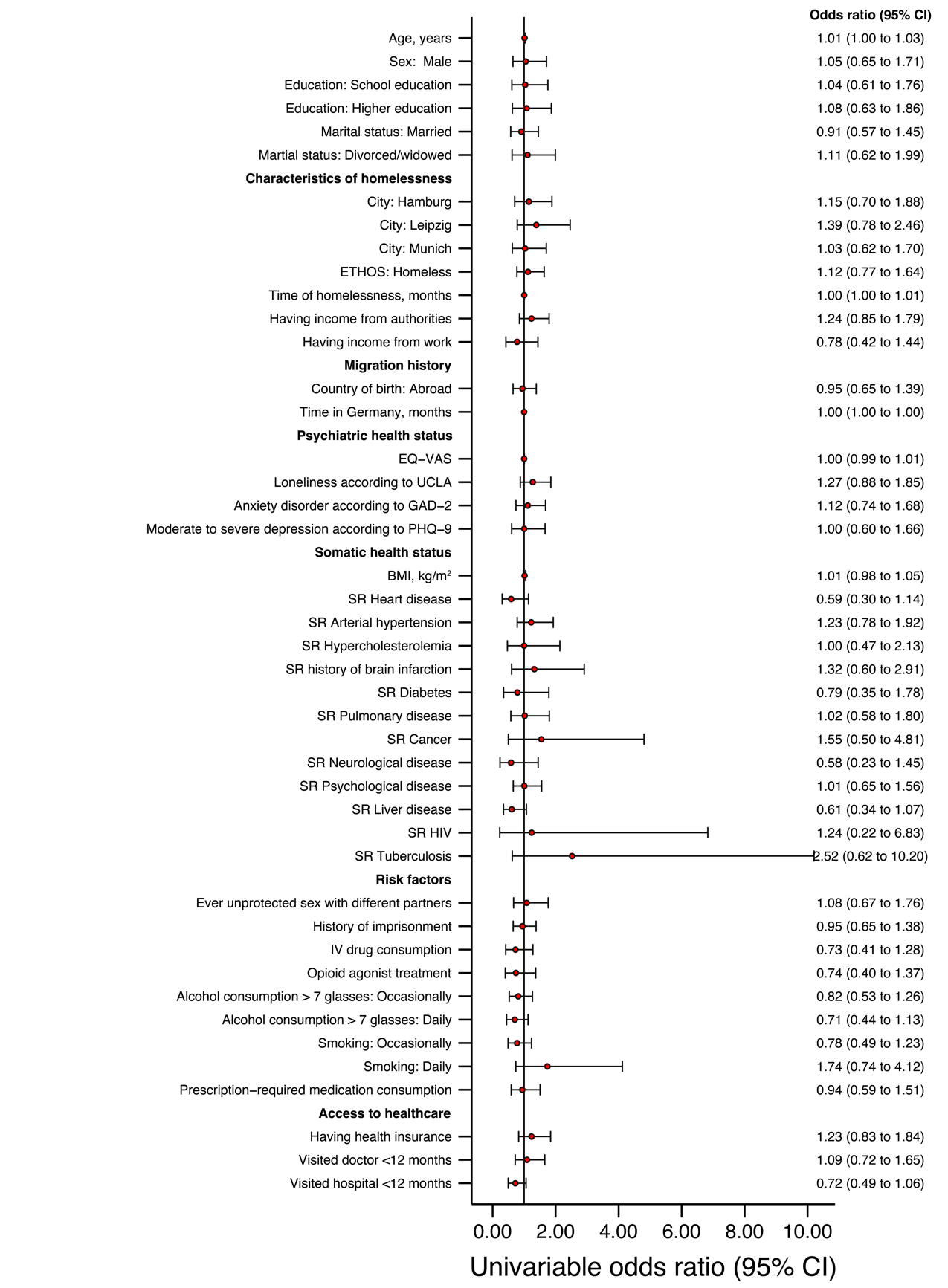
**Supplementary Figure 14.** Odds ratios and 95% confidence intervals with active or past HEV infection as the model-dependent variable are illustrated.

**Abbreviations:** ETHOS, European typology of homelessness and housing exclusion; EQ5D-VAS, standardised measure for health-related quality of life; UCLA-3, standardised questionnaire for loneliness; GAD-2, standardised questionnaire for anxiety disorders; PHQ-9, standardised questionnaire for depression; BMI, body mass index; SR, self-reported;, OAT, opioid agonist therapy.


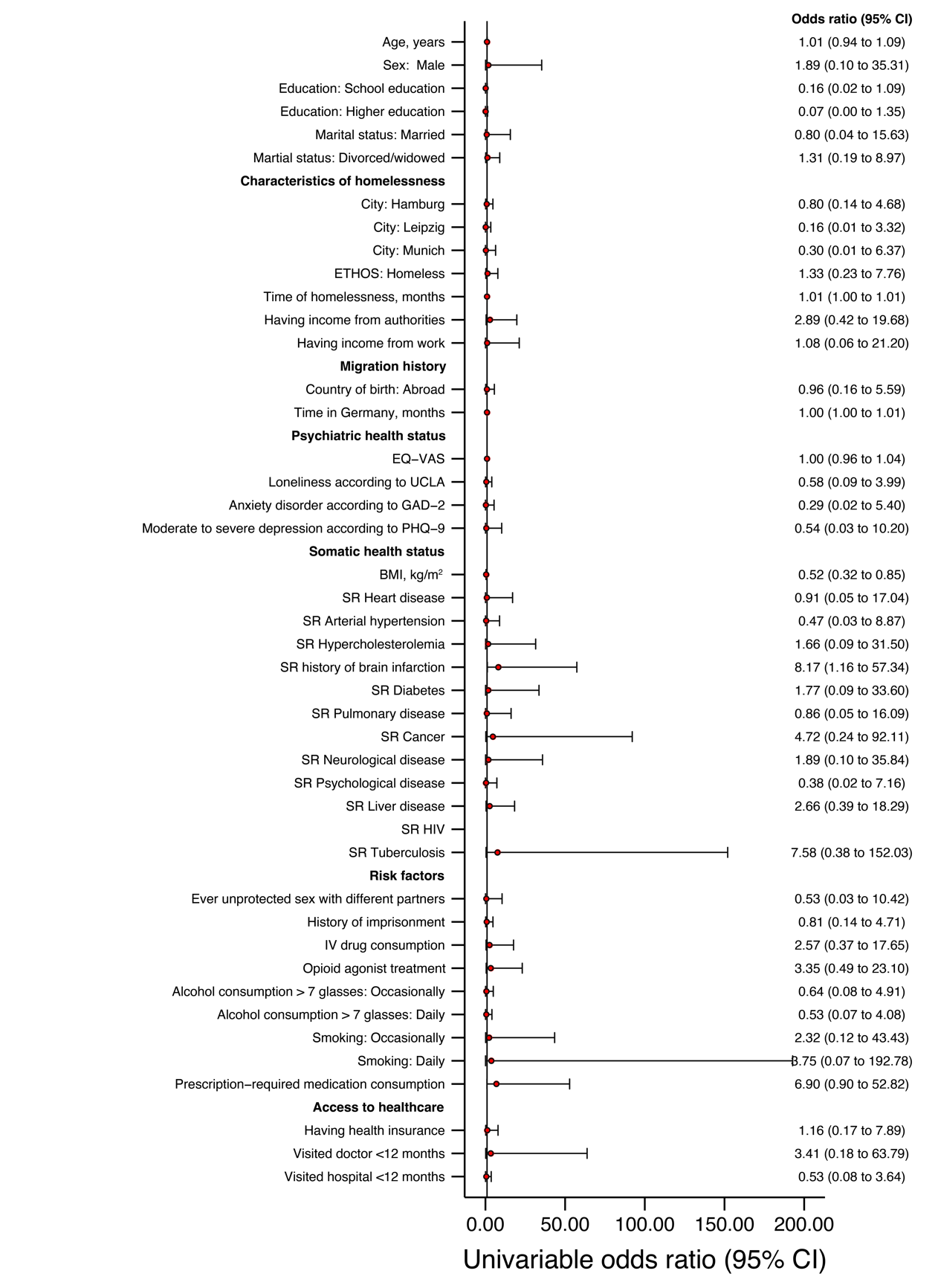
**Supplementary Figure 15.** Odds ratios and 95% confidence intervals with active HIV infection as the model-dependent variable are illustrated. Firth bias correction was applied. Self-reported HIV was excluded due to strong collinearity.

**Abbreviations:** ETHOS, European typology of homelessness and housing exclusion; EQ5D-VAS, standardised measure for health-related quality of life; UCLA-3, standardised questionnaire for loneliness; GAD-2, standardised questionnaire for anxiety disorders; PHQ-9, standardised questionnaire for depression; BMI, body mass index; SR, self-reported, OAT, opioid agonist therapy.

**Supplementary figure 16.** Directed acyclic graph (drawn using DAGitty(1)) on the causal relationship between imprisonment and hepatitis C infection.


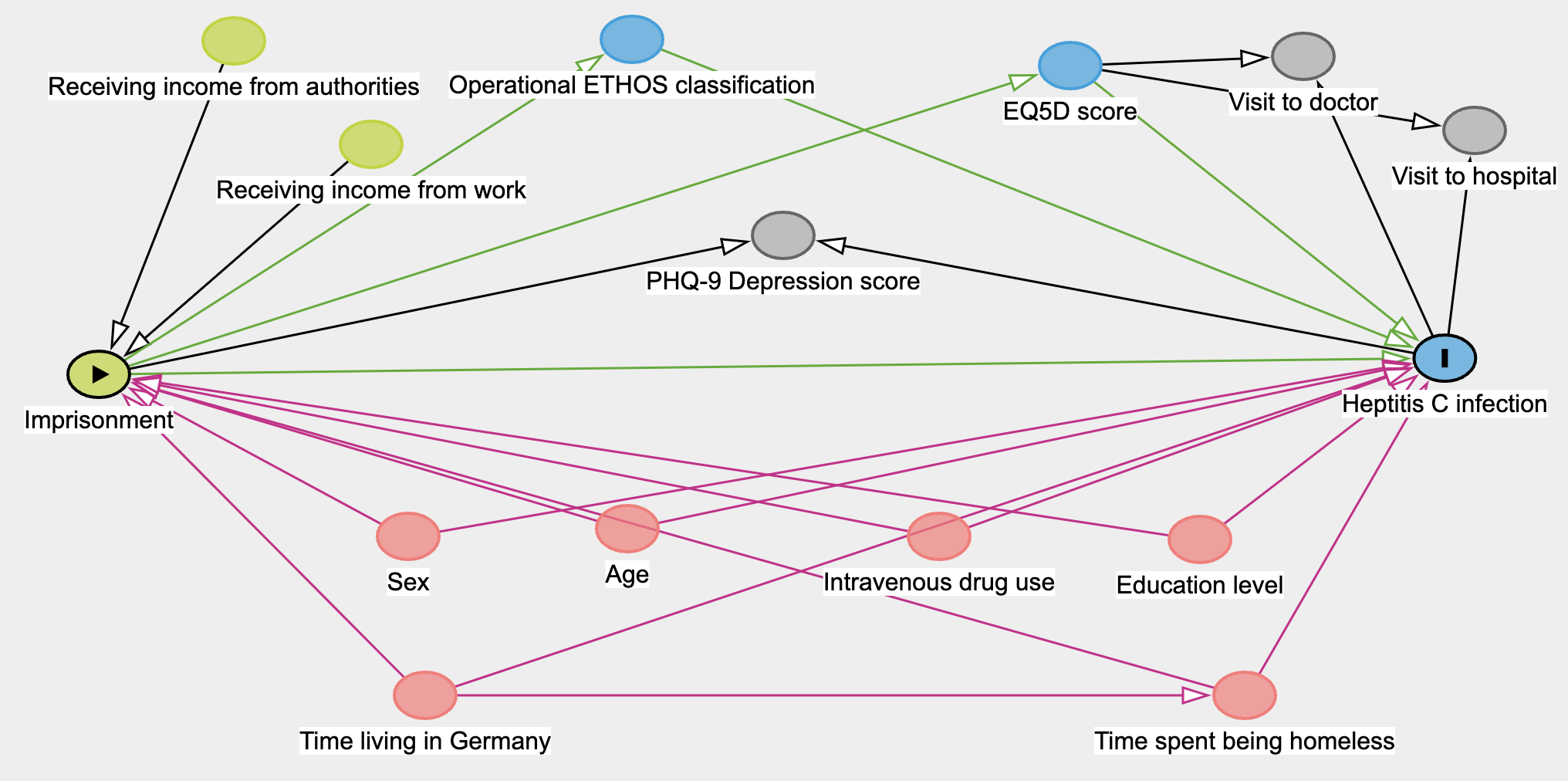


**Supplementary Figure 17.** Distribution of class assignment frequencies across 100 imputation datasets by latent class.**
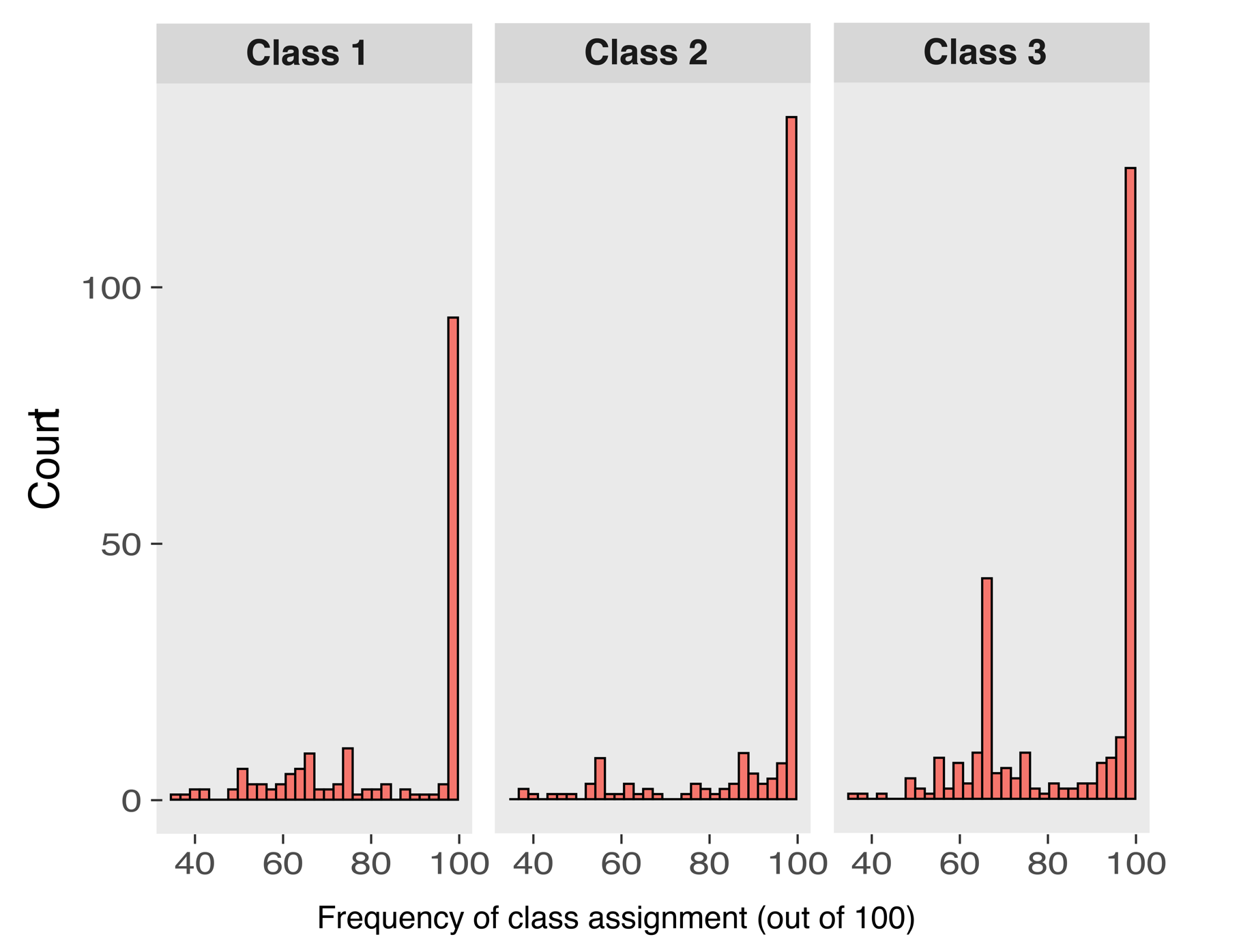
**

**Supplementary Material.** Key steps in latent class analysis.

Latent class analysis was used to uncover underlying, unobserved relationships between variables according to their distribution. Descriptive analysis $\chi^{2}$ tests were used to compare the proportion of categories within each variable against the outcome of hepatitis infection or immune status. To improve the robustness of the LCA, categorical variables were examined to identify any categories with a small number of observations. Categories with less than 10% of observations were merged with other clinically appropriate categories, with consensus within the research team. The distribution of continuous variables was examined using histograms and quantile-quantile plots.

Missing data in the dataset was investigated by examining the percentage of missing data in each variable and the missing data pattern across all variables and observations. Multivariate imputation by chained equations was employed to impute missing data. The imputation method was decided for each variable based on its data type and distribution. Imputed values were interrogated using tables and graphs to ensure that the generated values remained clinically plausible and to ensure agreement between imputed and observed values. One hundred imputations were performed, each imputation using a maximum of 30 iterations to generate 100 imputed datasets for LCA in the next step.

The approach to carrying out LCA suggested by Sinha et al. was generally followed in this study (2). First, variables were selected for LCA based on their plausible association with the hepatitis outcomes. Factors that are considered possible consequences of hepatitis were excluded from the analysis. To avoid spurious latent classes and poor convergence (3), collinearity between variables was investigated using the appropriate tests ($\chi^{2}$ test for binary or categorical variables, Pearson or Spearman’s correlation tests for continuous variables, t-test or Mann-Whitney U test between continuous and discrete variables). For continuous variables that do not follow a normal distribution, various transformations (e.g., log, inverse, squared) were explored to improve the distribution of the variable. Several assumptions were made in the LCA, these include: conditional independence of observed variables, mutually exclusive and exhaustive latent classes, and correct specification of latent class models. Then, LCA was performed on each of the 100 imputed datasets; 1 to 10 latent classes were explored for each dataset. Model fit and class separation were considered to determine the overall best-fitting number of classes. The Bayesian Information Criteria (BIC) and Akaike Information Criteria (AIC) were used to indicate model fit, while entropy was used to indicate class separation; these were compared between the classes and between imputed datasets. Supplementary Figure 1 shows the distribution of BIC, AIC, and entropy across the classes and imputed datasets. After the optimal number of classes was decided based on these metrics, the number of observations per class and the clinical plausibility of variable distribution were compared between the classes. Also, the distribution of variables between latent classes was examined to ensure that the distribution of a single variable did not determine the separation of latent classes. The optimal number of classes for the dataset was obtained when all the above proved satisfactory. Since the LCA would label the classes differently in each imputed dataset, the classes had to be relabelled to have the same meaning across imputed datasets; this is performed using the Jaccard index (4). Finally, the posterior probability of class assignment for each observation over all imputed datasets was used to determine class assignment for each observation. Supplementary Figure 17 demonstrates the variability in the posterior probability of class assignment within and between imputed datasets.

The final output of the LCA contains the class membership of each PEH individual. Everyone was assigned a class shared with other PEH individuals with similar sociodemographic and risk behaviour profiles. The distribution of variables between classes was examined using the appropriate descriptive analysis. The odds of hepatitis infection or immunity and the odds of HIV infection were compared between classes using logistic regression analysis.

**Supplementary Material.** Modelling results for the Latent class analysis (LCA).

LCA showed that a three-class model provided the best fit for this cohort, as indicated by model fit statistics, i.e. scree plots of Akaike information criterion (AIC) and Bayesian information criterion (BIC) suggested best latent class models at three classes, and that higher number of classes did not significantly improve fit statistics (Supplementary Figure 15). The scree plot of entropy suggested that class separation at three classes was better than a higher number of classes, thus making a three-class model the most appropriate latent class model based on these objective criteria.

The final class assignment for everyone was based on the most frequent class assignment across the 100 imputed datasets, while the class assignment within each imputed dataset was based on the highest posterior probability. The median frequency of individuals being assigned class 1 amongst the 100 imputed datasets was 98% (IQR: 93 to 100), 99·9% for class 2 (IQR: 93 to 100), and 99·8% for class 3 (IQR: 99 to 100) indicative of good model fit and high probabilities of a latent class assignment (Supplementary Figure 17).

**Supplementary Material.** Estimating the causal effect of imprisonment on HCV infection rates

To estimate the causal effect of imprisonment on HCV infection, the average causal effect was used as the estimand to provide an estimate of the average population effect by predicting the difference in expected outcome (i.e. HCV infection) as if everyone was imprisoned versus the expected outcome as if no one was imprisoned. Absolute risk difference was used to represent average causal effect. Absolute risk difference was estimated using augmented inverse probability of treatment weighted estimator, which uses a propensity score model to adjust for confounding between imprisonment and HCV infection. Logistic regression models were used to estimate the propensity score and the outcome. Directed acyclic graph (see Supplementary Figure 16) was used to represent the causal relationship between imprisonment and HCV infection, and confounding factors were selected accordingly. Age, sex, educational attainment, time of homelessness, time in Germany, and current intravenous drug consumption were included as covariates to adjust for confounding. Weights generated in the propensity score model were assessed to ensure reasonable weights with respect to sample size. Kernel density plots of the estimated propensity score were examined to ensure satisfactory balance of confounding factors.

**Literature**

1. Textor J, van der Zander B, Gilthorpe MS, Liskiewicz M, Ellison GT. Robust causal inference using directed acyclic graphs: the R package 'dagitty'. Int J Epidemiol. 2016;45(6):1887-94.

2. Sinha P, Calfee CS, Delucchi KL. Practitioner's Guide to Latent Class Analysis: Methodological Considerations and Common Pitfalls. Crit Care Med. 2021;49(1):e63-e79.

3. Tarka P. An overview of structural equation modeling: its beginnings, historical development, usefulness and controversies in the social sciences. Qual Quant. 2018;52(1):313-54.

4. P. J. Distribution de la flore alpine dans le Bassin des Dranses et dans quelques regions voisines. Bulletin de la Société Vaudoise des Sciences Naturelles 1901(37):241-72.
